# Constructing *Centimeters*: Emanuel Friedman’s Cervimeter and the Dilatation-Time Curve*

**DOI:** 10.1353/bhm.2025.a963726

**Published:** 2025-01-01

**Authors:** Rebecca L. Jackson

**Keywords:** history of measurement, history of obstetrics, obstetric instruments, measurement units, bureaucratization, measurement methodology, biomedical validation

## Abstract

In 1954 Emanuel Friedman created a new dimension for measuring labor—change in dilatation rate over time—allowing the birthing body to participate in defining what it meant for labor to be “arrested.” Yet in constructing a “normal” standard curve of dilatation-over-time for guiding labor decisions and constructing a measuring instrument (the “cervimeter”) to evidence the shape of this curve, Friedman unintentionally enabled a new dimension of labor to emerge: centimeters of dilation, today read as the state of labor progress. This article examines an oral interview with Friedman, the raw data from his first study, and his published research to show how the cervimeter reified centimeters as an “objectively” measurable interval-scale unit (rather than representing an ordinal approximation felt by hand) and enabled the transformation of Friedman’s curve from a graphical tool meant to conform *to* women into a tool used to conform them.

Since the 1950s, the Friedman curve has been used to define the normal length and duration of labor stages … the resultant sigmoid curve that Friedman divided into latent, active, and transition phases has been used by all maternity care providers in the United States to identify women who have labor dystocia … the Friedman curve is not an accurate reflection of the true course of labor.… Use of the Friedman curve today results in higher rates of dystocia diagnoses and cesareans than is necessary.^1^

Due to high rates of caesarian sections in the United States,^2^ there has been recent pushback against the use of cervical dilation thresholds for clinical decision-making. By “cervical dilation thresholds,” I refer to the minimal number of *centimeters* we expect the cervix to be dilated given the number of hours in active labor. Obstetric decisions related to triage, vigilance, diagnosis, as well as hormonal and surgical interventions are often wholly or partly justified based on these thresholds. The “active phase” of labor itself is defined by a dilation threshold, beginning at 4 cm of dilation.^3^ Certified nurse-midwives Tekoa King and Whitney Pinger, quoted above, trace the historical misuse of cervical dilation back to Emanuel A. Friedman, a U.S. obstetrician and researcher. Friedman’s most renowned contribution is his eponymous curve of dilatation-over-time, a function that tracks the diameter of the cervix (in centimeters) during the course of labor. King and Pinger believe the “Friedman curve,” the standard from which dilation thresholds are derived, is “not an accurate reflection of the true course of labor” and is responsible for the overdiagnosis of difficult/obstructed labor (dystocia) which requires intervention. Unnecessary interventions such as C-sections can lead to worse maternal and fetal outcomes and increased risks for future births.^4^ A deficit of safe vaginal births may even affect gut health at the population level by interrupting the transfer of valuable vaginal microbiota from mother to infant.^5^

King and Pinger are not alone in regarding Friedman as the historical origin of dilatation data misuse. In 2014, the American College of Obstetricians and Gynecologists (ACOG) along with the Society for Maternal-Fetal Medicine (SMFM) released their reformative report on the future of labor management safety. In this report, they linked Friedman’s earliest work to the dilation thresholds used today that have led to overdiagnoses of arrested labor and failure to progress: “*Based on Friedman’s work*…*active phase arrest traditionally has been defined* as the absence of cervical change for 2 hours or more in the presence of adequate uterine contractions and *cervical dilation of at least 4 cm*. … The Consortium on Safe Labor data [which suggests 6 cm as a better threshold], rather than the standards proposed by Friedman, should inform evidence-based labor management.”^6^ Similarly to King and Pinger, the ACOG and SMFM stated that the Friedman standard has stood in the way of evidence-based care and suggested a more liberal standard be embraced (albeit one still tied to a dilation threshold). Given this, these organizations and authors may be surprised that their position on labor management is actually far more generalized and stringent than the one that Friedman held (and currently holds).

Friedman’s position in this debate has a much more complex and dynamic history than is suggested by the above references to “Friedman” as an eponym, a relic of obstetrics past. In a 2016 interview, Friedman railed against the very idea of setting *any* predetermined thresholds for phases of a woman’s labor.^7^ This article traces how Friedman’s early research actually turned the eyes of medical practitioners back toward the laboring body and challenged standards of labor care that were based solely on time limitations without serious regard to physiological processes. Friedman’s original goal was to create an individualized standard based on the rate of dilatation of the individual woman. His method of graphically recording dilatation data required continual attention to each labor as a unique process, unfolding in real time. His intention was not to establish a single set of predetermined thresholds in centimeters of dilation, as we see in partograms (labor charts) today.

Particularly, this article clarifies the role of Emanuel Friedman’s “cervimeter” in his early work. The cervimeter was a pair of modified forceps: curved metal bars twenty-five centimeters long, articulated at the midpoint, with eight-millimeter serrated “bulldog” clips welded to each end for clamping opposite sides of the cervical rim. A metal ruler was attached to one of the handles, calibrated to reflect the distance between the two clips in *centimeters* (see Figure 6 below).^8^ This object, which Friedman called an “excellent academic tool” for measuring cervical dilation, was the “objective device” that validated the manual method he used to arrive at his dilatation-time curve.^9^ The cervimeter confirmed the S-shape of the curve, legitimizing Friedman’s treatment of his data as true *centimeters*-over-time. In the process, the cervimeter effectively reified *centimeters* of dilation as used today, with strict thresholds that treat each level of centimeters as equidistant points (“interval-scale” units). This diverged from previous treatments of dilation quantities, which were represented by merely approximate, ordered values (“ordinal-scale” units, for example, “two fingers” representing a smaller opening than “three fingers”).^10^

In Friedman’s own accounting of this history two decades later, he leaves out the role of the cervimeter completely.^11^ Despite this, this instrument was a rhetorically important source of evidence for convincing skeptics of the shape he hypothesized for his dilatation curve, which he considered his main contribution to the understanding of labor—albeit limited to the research context. Friedman’s cervimeter, in many respects, failed to offer any substantial benefits in the clinical context and is now a forgotten relic of the past. Friedman himself never intended for it to serve as a daily clinical substitute for the manual method (still used today) and warned others against such efforts.^12^ Yet the evidentiary role this instrument played in Friedman’s argument had a lasting impact on clinical standards. The legacy of the cervimeter is “centimeters” of dilation, as we now understand them: as equidistant units of not just dilatation, but labor progress. This is the legacy, in the wake of evidence that has undermined the role of *centimeters* as a clinically significant unit of dilation, which may now need to be undone.

While this is a story about Friedman’s cervimeter, it is also a story about the underlying physicality of measuring practices in the high-stakes, temporal, increasingly bureaucratized landscape of American obstetric practices from the 1950s to the 1970s.^13^ In this period, the advantages of sensory reference objects give way to the advantages of paper technologies that could be used in labor management and recordkeeping. The following section offers examples of some standard (and nonstandard) “unit-objects” that predated the cervimeter. These sensory reference tools enabled practitioners to construct individual records of dilation during labor that could then be used to form aggregated data. While the aggregated dilation data were originally used to generate general graphical comparisons to evaluate the efficacy (or lack thereof) of certain labor interventions, the graphical picture verified by the cervimeter (the Friedman curve) eventually took on a life of its own as a template for individual labors to follow in real time.^14^ The section preceding the conclusion discusses two paper technologies that came into use after this transformation, namely “partograms” for visually tracking labor progress and worksheets for auditing patient care. These objects, in addition to the cervimeter, contribute to a fuller understanding of how the artisan practices of labor management of the early twentieth century gave way to the bureaucratization of birth that would take shape in the latter half of the century.

Despite the temporal and geographic focus of this article being quite narrowly focused on obstetric materials and practices in the mid-twentieth-century United States, broad connections can be drawn. Because Friedman’s research is best understood within broader debates about cervical dilation units, I begin by describing his European influences and the ways in which he departed from them. The story that follows then primarily centers on Friedman’s research conducted at Sloane Hospital for Women in New York in the 1950s and 1960s. Yet what occurred during this period had rippling international impacts in the decades to come (and still today). This has been demonstrated by Jacqueline Wolf’s account of the cesarean section operation in the United States, which focuses on the roles of obstetric technologies in shifting public and medical perceptions of risk over the course of the twentieth century. She points to the Friedman curve as the primary technology that broadened the definition of dystocia (stalled labor) and thus increased the number of dystocia diagnoses that led to cesarean sections. This in turn led to an increasing rate of cesareans performed in the United States and beyond (e.g., England, Brazil, even the Netherlands, though their rate is still comparatively low), bolstered by other technological changes that increased the public perception of nonintervention as inherently risky.^15^

Yet Wolf’s account treats Friedman’s curve as a relatively static standard that was “quickly and widely adopted,” omitting the material and rhetorical role of the cervimeter entirely from Friedman’s project.^16^ In my account, the dilatation-time curve is not treated as self-evident to Friedman’s contemporaries. Similarly, *centimeters* of dilation are not the obvious or inevitable unit for characterizing “normal” labor progress. I focus on the difficulties facing the construction of this unit and on the agency of physical and mathematical tools in enabling cervical dilation status to become abused as a proxy for labor progress. The material history of labor progress quantification is deserving of much more thorough treatment by historians; this article may be among the first to examine twentieth-century cervical measurement closely, a subject already of interest to medical audiences.^17^ For historians of medicine, the role of instruments and paper technologies in creating a seemingly neutral unit for cervical dilation, *centimeters*, serves as a reminder that measuring units and interpretations of “precision” can be probed for how they align with institutional, rhetorical, and bureaucratic priorities.^18^

## “Object”-ive Cervical Measurement Before Friedman: Fingers, Coins, Watches, and Centimeters

In current labor management practices, cervical dilation in centimeters is used to reference the state of labor progress (e.g., “You’re 4 cm along.”). Many high-stakes care decisions hinge on dilation estimations, which obstetric practitioners perform by manually palpating the cervix (since it cannot be observed visually). The cervix is the passage between the uterus and the vaginal canal which the fetus traverses in exiting the womb. The dilation of the cervix refers to the diameter of the external os, an orifice surrounded by firm tissue which gradually softens and recedes during labor to allow the fetus to enter the vaginal canal. For natural (vaginal) births, this occurs when the cervix is fully dilated, conventionally thought of as 10 cm. Yet we have not always thought of cervical dilation from 0–10 cm as the scale for labor progress.

Emanuel Friedman’s “cervimeter” instrument, developed in 1956, is central to my account of how a theoretical idealization (the diameter of the cervix in *centimeters*) transformed into a representation of the dynamic physiology of the birthing body (labor progress). Yet Friedman was not the first to quantify the cervical os, nor did his cervimeter represent the first time that practitioners employed tools in making cervical estimations. Other objects shaped this historical progression by their presence (or absence) long before Friedman. In the early twentieth century, familiar handheld items (such as coins, rings, watches, or even fingers) were used as units for estimating dilation, which demonstrates that measuring units were not thought of as mere abstractions. These “unit-objects” operated as useful sensory references.

In 1920s Germany, discussion about dilation measuring units and nomenclature was active and public. The unit system that had been in place, based on marks (which included one-, two-, three-, and five-mark coins as units for describing smaller dilations), was becoming obsolete due to the influx of fiat currency and resulting scarcity of the coins as reference standards. One proposal was that these once-familiar units should be translated to the approximate number of centimeters the objects spanned (see Table 1).^19^ Yet this solution was seen as irrelevant to solving the true problem at hand: an absence of accessible, physically meaningful reference standards. Wilhelm Liepmann argued, “Anyone who has had students determine the size of an object in centimeters over many years of instruction knows how different and uncertain the specifications are for objects that the student sees every day, let alone those he can only feel. The path of centimeter determination is therefore not practicable.”^20^ Instead, Liepmann proposed an entirely new set of familiar handheld objects that were “so well known that every student and every midwife has a certain idea about them” (see Table 2).^21^ These objects, or metal disks created to be approximately the same diameter in centimeters, were proposed as tactile training tools for the sense of touch to be able to correctly identify these distances. Here we see that “centimeters,” too, were first and foremost regarded as a set of objects (metal disks) that needed to fulfill the obligation of creating a reliable connection between mental objects and sensory estimations. Not all centimeter values appear in this system (e.g., 7 cm is missing from both tables), as “centimeters” operated as merely a mediating tool in an ordered system of more meaningful objects.

Among unit-objects, “centimeters” was one of many choices, with advantages and disadvantages—and was thought to be most advantageous as a translation of a system based on more sensible units. Decades later, Emanuel Friedman’s use of “centimeters” as truly metric *centimeters*, a unit abstracted away from the senses, was a departure from previous obstetric practices and had to be defended with evidence. Just as it was far from inevitable that cervical dilation would ever be measured with an interval-scale unit (*centimeters)*, it was also not inevitable that dilation itself would take on the role of proxy for labor progress as it did in the latter half of the twentieth century. Before cervical dilation was reduced to a diameter (an abstract Euclidean distance) by Friedman’s cervimeter, the thinning cervical os was regarded as something much more material.

In early twentieth-century American obstetrics, the cervix was seen as a physical constraint on a skillful childbirth—a barrier that could be unnecessarily torn in the midst of the physician’s attempts to advance labor progress too quickly. Physicians were warned not to encourage a woman to push too early “before full dilatation,” as this could cause tearing or lead to unnecessary operations.^22^ Such lacerations “were a symbol of the attendant’s skill,” or lack thereof, and thus important to avoid so that the physician did not “acquire the reputations of tearing his women.”^23^ The cervix was a corporeal limitation to be respected, for the sake of medical ethics and personal reputation.

By the mid-twentieth century, some European obstetricians had begun tracking cervical dilation to test general research claims, such as whether breaking the patient’s water had any impact on speeding up labor.^24^ Yet even for this academic use, tracking dilation alone was criticized as too broad of a brush to use for arriving at clear conclusions.^25^ When recording dilation data in order to publish their experimental results, obstetric researchers were just as likely to use a nonuniform, ordinal unit like “fingers” in such studies, such as “1–2 fr.,” “5 fr.,” “small palm” (which Friedman took to be about 6 cm), “large palm” (about 8 cm), with unequal distances between the categories.^26^ While we might marvel at such imprecise recording, for the purposes of assessing the whether breaking a patient’s water has an impact, these units do not seem all that unreasonable. If the difference between “intervention” and “no intervention” is so little that it requires a more precise measuring unit than that which the clinicians regularly use in their practice, especially in light of the general variance among individual labors, it is unlikely to be considered a clinically significant difference. Even when “centimeters” were nominally used in these studies, this was likely a translation from units derived from familiar unit-objects (as described in the section above). Friedman’s goals, as will be shown, required “centimeters” to be more than a mere translation of ordinal units. In the process, cervical dilation became something that *centimeters* could represent: a diameter.

## Emanuel Friedman’s Curve and the Birth of “Normal” Labor Progress

Once Emanuel A. Friedman became a junior resident at Columbia-Presbyterian Medical Center in New York, one of his professors, Virginia Apgar, had a question for young Friedman to pursue: Is there a way to determine whether caudal anesthesia has any impact on the course of labor?

There is a deep irony in this mid-twentieth-century question of how to measure the effect of anesthesia on labor. In the century prior to Friedman’s entrance into medicine, an important way physicians could gauge labor progress was to monitor the changes, in quantity and quality, in a birthing woman’s pain.^27^ Some physicians gave this as a reason for why they hesitated to employ anesthesia in the mid-nineteenth century to dull labor pains; Dr. Charles F. Meigs of Philadelphia “relied upon women’s painful contractions to help him determine labor’s progress and believed that their inhibition would make him a less effective birth attendant.”^28^ Women’s pain was their individual voice, at times literally, in how far along their labor was and how it was progressing in real time.

The use of anesthesia in childbirth had become common practice by the early twentieth century, and yet maternal mortality had remained high, beginning to fall only in the decade before Friedman entered his residency.^29^ As such, women’s pain was obscured and their screams interpreted as reasons for increasing sedation and reducing consciousness.^30^ There was no certainty whether this reduction in pain was worth the possible dangerous effects of slowing labor, if “drugged women were less effective at pushing the baby out.”^31^ The use of some kind of anesthesia, applied in the hospital context, became the standard. Beyond that, all other standards—the type and timing of anesthesia administered, the variables of each individual woman that were relevant or predictive—were up for grabs and difficult to evidence. It was amid this backdrop that Virginia Apgar asked Friedman to find an objective measure for labor, at a time when women’s individual voice in their own labors was at an all-time low.

Whereas European researchers had used dilation for tracking the effects of individual interventions (such as stripping amniotic membranes to speed up labor), Friedman’s goals grew to be much broader. Friedman became determined to find a way to “measure labor objectively.”^32^ He saw previous practitioners as having ignored “the task of establishing a ‘norm’ for the course of labors, with which comparisons may be made,” and he sought to do so.^33^ More than to simply form “before/after” comparisons for a given intervention as it was imposed on subjects, Friedman’s recording of cervical dilation over time was out of a desire to understand the nature of “normal” labor. He wished to form a firm basis for claims of *what would have happened* “without” and “with” any given intervention.^34^

Friedman began by recording all of “the major observable events that occur during labor,” serially in graphic form, including data about contractions (“force, frequency, and duration”), the “descent of the presenting fetal part” (the baby’s “station”), and the cervical effacement and dilatation.^35^ Friedman’s use of the word “observable” here seems to denote “graphically recordable,” as certainly there were many other observable events in the course of labor, related to a patient’s breathing, fetal and maternal stress, cervical ripeness (consistency), and vocalized information. From the visualized data that Friedman did compile, he noticed no meaningful pattern from the frequency or duration of contractions, but a strikingly consistent S-shaped curve in the progress of cervical dilatation.^36^

This “sigmoidal curve,” as he called it, was characterized by a slow dilatation rate up to a point, which he later termed the “latent phase of labor,” followed by the “active phase,” wherein the cervix dilation rate increased quickly, then remained at a consistent rate of increase approaching 10 cm. After subsequent studies that purposefully sought to verify and establish this trend, he identified what appeared to be a “deceleration phase” of slower dilation rate approaching delivery.^37^ Thus was born the “Friedman curve,” which he called the “dilatation-time function,”^38^ and which eventually became the standard for labor decisions for over fifty years.

Beyond establishing a control for studying the effects of interventions on labor on the level of aggregated results, Friedman also thought his curve could be used “to provide a realistic tool for the study of individual labors, in progress, by obstetricians outside of university hospitals.”^39^ Friedman’s desire for a standard for “normal” labor to also act as a standard for clinical *practices* was just one manifestation of a widespread effort to fill the gaping vacuum of standards in labor management more generally. Friedman entered American obstetric medicine during a tumultuous time; little was established with consensus, the only constant being the discipline’s unique commitment to criticizing itself.^40^ Practitioners complained that labor management practices were rooted in too much superstition or tradition, along with “accepted facts … based on the study of an extremely small number of patients.”^41^ Long-standing assumptions, such as a woman’s advanced age posing an inherent risk to her first labor, were in the process of being seriously questioned if not debunked.^42^ Friedman lamented the bias in care that occurred regularly to older first-time mothers, whose labors were more likely to be subjected to surgical interventions, “often with dubious indication.”^43^

This commitment to self-doubt and self-examination left a relative vacuum of shared standard practices in the field, and a free for all in terms of which patient attributes may be most relevant for predicting and managing birthing outcomes. Yet what was clear to young Friedman during his residency was that the reigning paradigm of simply keeping track of the number of hours and intervening on lengthy labors was no longer acceptable: “In light of the present-day knowledge, evaluations based largely upon study of total labor or total first stage duration lack in critical accuracy.”^44^ Friedman wanted to offer a picture of labor that could provide insight into the ways that uncomplicated labors behaved that differed from complicated labors. Unlike previous guidance based solely on number of hours in labor, Friedman’s data were based on the dynamics of the birthing body.

**Figure 1:**
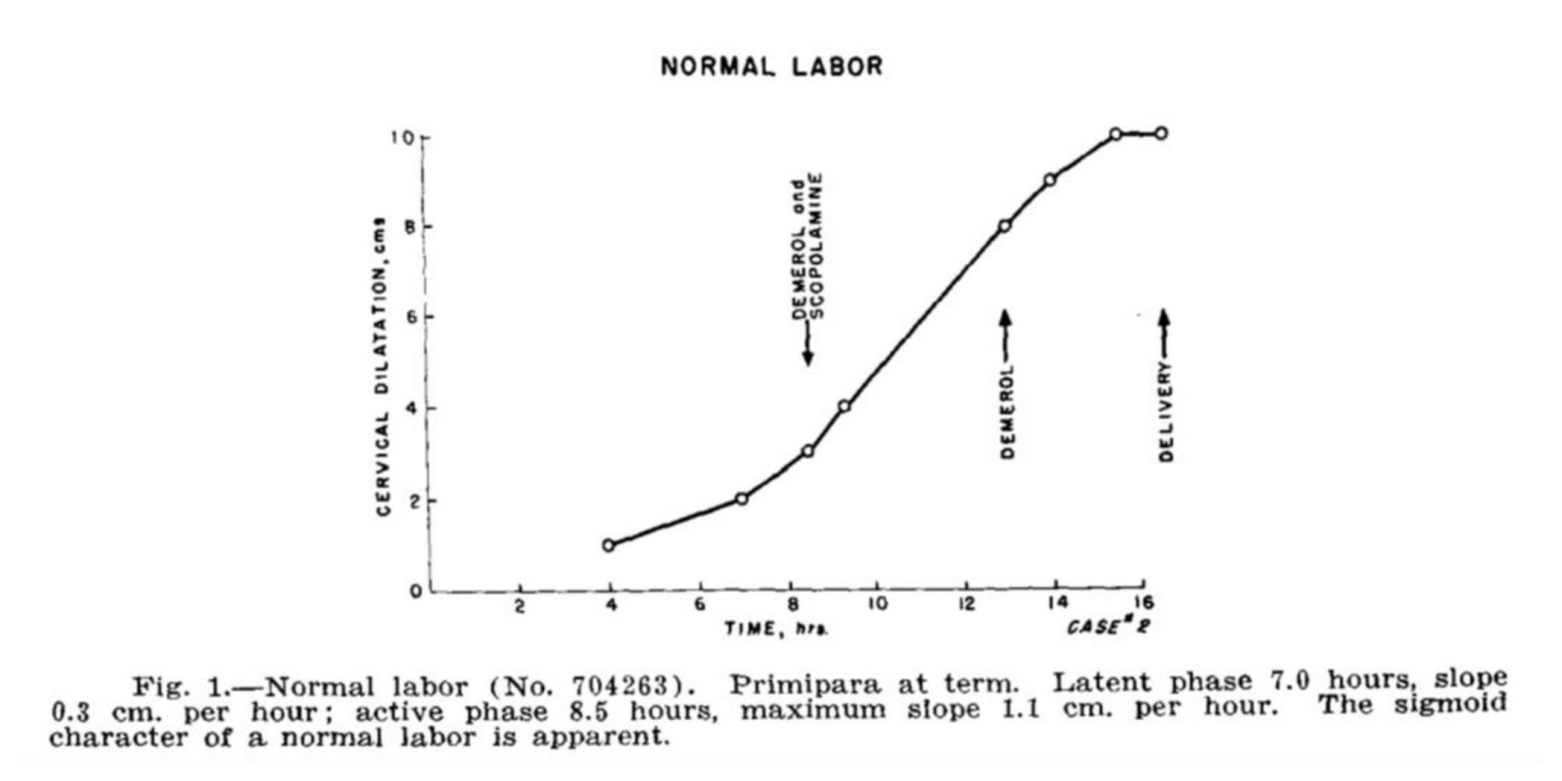
Emanuel Friedman’s Sigmoidal Dilatation-Time Curve. Source: Emanuel A. Friedman, “The Graphic Analysis of Labor,” *Amer. J. Obstet. Gyn*. 68, no. 6 (1954): 1568–75.

For his first formal study, Friedman looked only at first-time labors, one hundred women in total.^45^ First-time births could be especially long and arduous, even without complications; Friedman may have had this in mind, or may have merely wished to eliminate one variable that could confound his results. Friedman constructed this picture of normal labor by graphing cervical dilation data as points on a plane and connecting these dots with straight lines. One particular labor (“Case 2,” as seen in Figure 1) is selected to act as representative of the ideal. He explained that the curves of labors without complications were similar in shape, but did not display the shape of individual labors for the reader. Unlike previous dilation graphs, which were sometimes messy, with multiple lines and/or shaded areas depicting the range of values collected,^46^ Friedman’s graph was pristine and neat, a single line describing the course of normal labor. Based on this ideal shape, he defined four phases that occurred from the onset of labor until the moment before the fetus passes through the vaginal canal: the latent, acceleration, steady, and deceleration periods. Together, these formed the first stage of labor. The second stage of labor involved the expulsion or removal of the baby, which Friedman decided was outside the scope of his study. While several important and high-stakes decisions had to be made in the second stage, such as whether forceps or surgical procedures may be needed, the progress of this stage had no coordination with cervical dilation, and thus “its management is left as a clinical art.”^47^

The onset of labor was taken to be when regular contractions had begun (presumably, in contrast to sporadic and nonrhythmic contractions that do not increase in strength, i.e., “Braxton Hicks contractions”). The first phase, the “latent period,” then takes place until cervical dilatation is “appreciable,” but growing no faster than linearly, until 2 or 2.5 cm. He provided summary statistics for this period: mean slope of 0.35 cm/hr, with a minimum and maximum of 0.0 and 0.86 cm/hr respectively. The mean number of hours spent in this period was 7.3, with a large range of 1.7 to 15.0, with a standard deviation of 5.5 hours. Given this huge variance in length of the latent period, he cautioned that diagnosing “primary inertia” (arrested labor) during this period is dubious, noting that the length of this phase had no bearing on the future course of labor, according to his statistical analysis. He advised leaving this first phase out when calculating the total length of the labor and considered phases 2 through 4 to be the true “active phase” of labor, the length of which was more likely to be informative. He did note, however, that latent periods that extended beyond fifteen hours (his observed maximum) may be cause for alarm.^48^

The second phase, the “acceleration period,” was signaled by a rapid change in the slope of the dilatation curve. The rate itself begins continuously increasing (the second derivative becomes positive). When the maximum slope has been reached, the “steady period” begins and the curve becomes merely linear again (also referred to as the “phase of maximum slope”). The number of hours of this phase varies greatly. He viewed this third phase as “most important” and stated the effects of “interference”/interventions are most easily observed (as deviations from the linearity). He noted that this period extends from “3 or 3.5 to 8.5 or 9 cm.”^49^ The first stage of labor concludes with the “deceleration period,” wherein dilatation slows its growth and gently declines toward a flat slope as full dilatation is reached. This fourth and final phase is analogous to the second “acceleration phase,” but the dilatation rate calms rather than climbs (see Figure 5 below for Friedman’s later depiction of these phases on a composite curve).

Overall, a change in the rate of dilatation is what defines these periods. He also provided general windows for the state of cervical dilation at which the first and last of these phases may begin or end as well as summary statistics for how long they lasted and what slopes of change were observed. He concluded that the “main variation appeared in phase three,” or the steady phase, regarding both slope and length in hours.^50^ His decision to separate these phases piecemeal, rather than treat the labor curve as a whole, reflects a mere convenience of calculation; he explained that to treat them as one curve would be mathematically more complicated.^51^ Friedman’s background in mathematics shows, particularly in his footnotes, where he discussed and then dismissed a possible statistical transformation that would take the sigmoidal curve and transform it into a straight line; he noted that using this transformation would require an assumption of symmetry in the sigmoidal curve, which was not warranted by his data.

**Figure 2:**
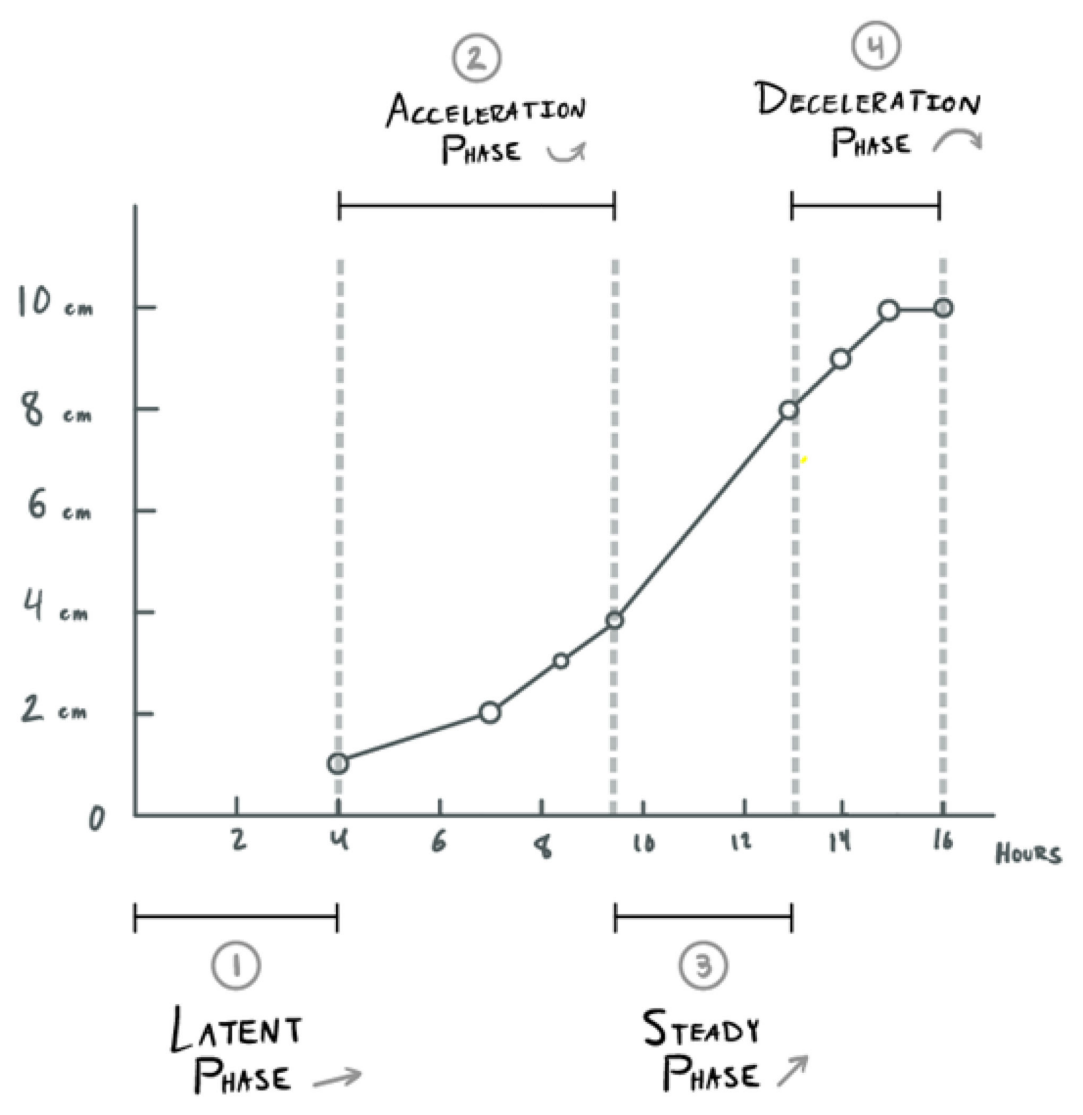
Friedman’s Four Phases of the First Stage of Labor (diagram mine).

**Figure 3:**
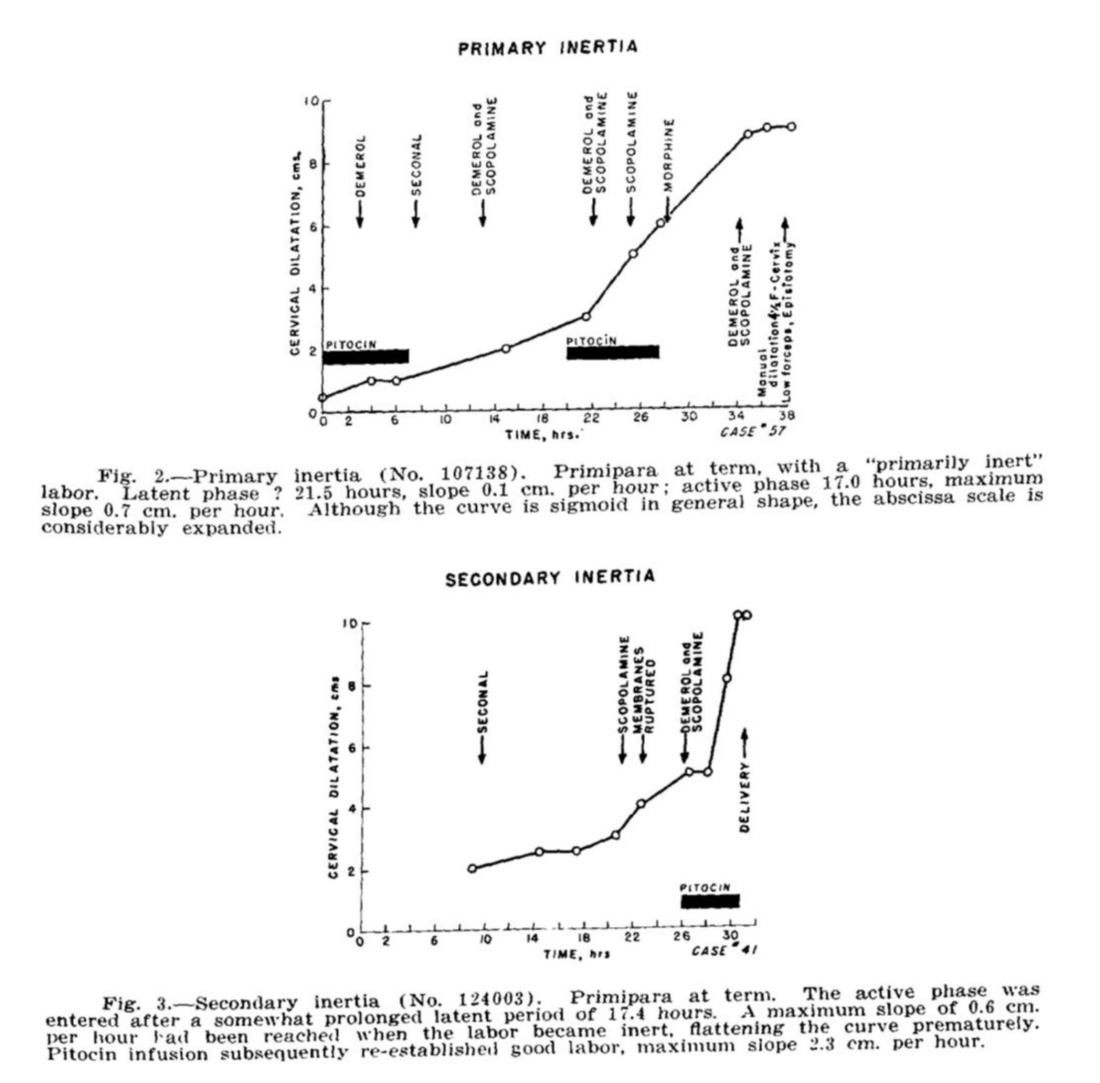
Friedman’s “Case 57” (exemplifying “primary inertia”) and “Case 41” (exemplifying “secondary inertia”). Source: Emanuel A. Friedman, “The Graphic Analysis of Labor,” *Amer. J. Obstet. Gyn*. 68, no. 6 (1954): 1568–75.

With this picture of normal labor, Friedman was able to define “abnormal” labor by comparison. He defined two kinds of labor “inertia” (stagnation), primary and secondary: “Primary inertia [arrested labor], redefined, is detected by a low overall slope, cervical dilatation occurring quite slowly, but nevertheless progressing along a normal sigmoid curve ([Friedman’s] Fig. 2). Secondary inertia is reflected in a deceleration of the slope prior to that expected (i.e., before 8.5 cm.), the preceding portion of the curve having been normal ([Friedman’s] Fig. 3).”^52^ He considered this standard for “primary inertia” to be an improved redefinition, based on slope of dilatation rather than simply based on length of labor, and allowed for diagnosis only after the onset of appreciable dilatation increases. This new definition would hopefully avoid misdiagnoses of labor arrest during the latent phase, wherein great variance in length had no impact on the rest of labor. He defined “secondary inertia” as the premature slowing dilation during the linearly growing “steady phase.” Friedman observed, “The flattening of the curve prematurely is readily detected and should alert the obstetrician to seek the cause… This prompt detection of arrested (or arresting) labor should prove of considerable value.”^53^ He displayed two patients’ individual graphs as prototypical cases of these two types of labor “inertia.”^54^ Just as “normal labor” was presented as a single curve, even the “abnormal” cases were given only one representative example with a single simple trajectory, listed as “Case 57” and “Case 41,” respectively (Figure 3). His study was observational, with no intentional influence on which interventions were to be performed or not performed during each labor. Thus, he notated these two cases with the drugs that were administered, showing graphically apparent “reactions” from the patient’s cervical dilatation rate.

It cannot be emphasized enough how important cervical dilatation *rate*, rather than the state of cervical dilation at any given point, was for Friedman. He characterized his goals thus: “What we have done is define a new dimension (slope), viewing labor as a dynamic process, setting time limits solely on the bases of previous activity, and, finally, demonstrating what may be expected of normal labor.”^55^ Focusing on rate changes allowed for each labor to have unique lengths of different phases, and unique rates of dilation during these phases, while still having a way to tell if something was going “wrong”: deviation from the rate established. While it may seem that his graphs are overly simplistic and leave out detailed information (e.g., we may wonder about all the other patients’ dilatation curves), displaying variance among patients was not necessary for his primary goal. That every labor was different came as no surprise to Friedman; this was the assumption and the problem. If even uncomplicated labors had large variance in their length and course, how could one tell normal variance from an abnormal and truly significant deviance? This is the purview of statisticians and the purpose behind statistical testing, like the kind that Friedman employed in his analysis. Using a continuous interval unit, centimeters, to record a measurable indicator for labor progress, he constructed a way for labors to still be tractable while still being individual.

**Figure 4:**
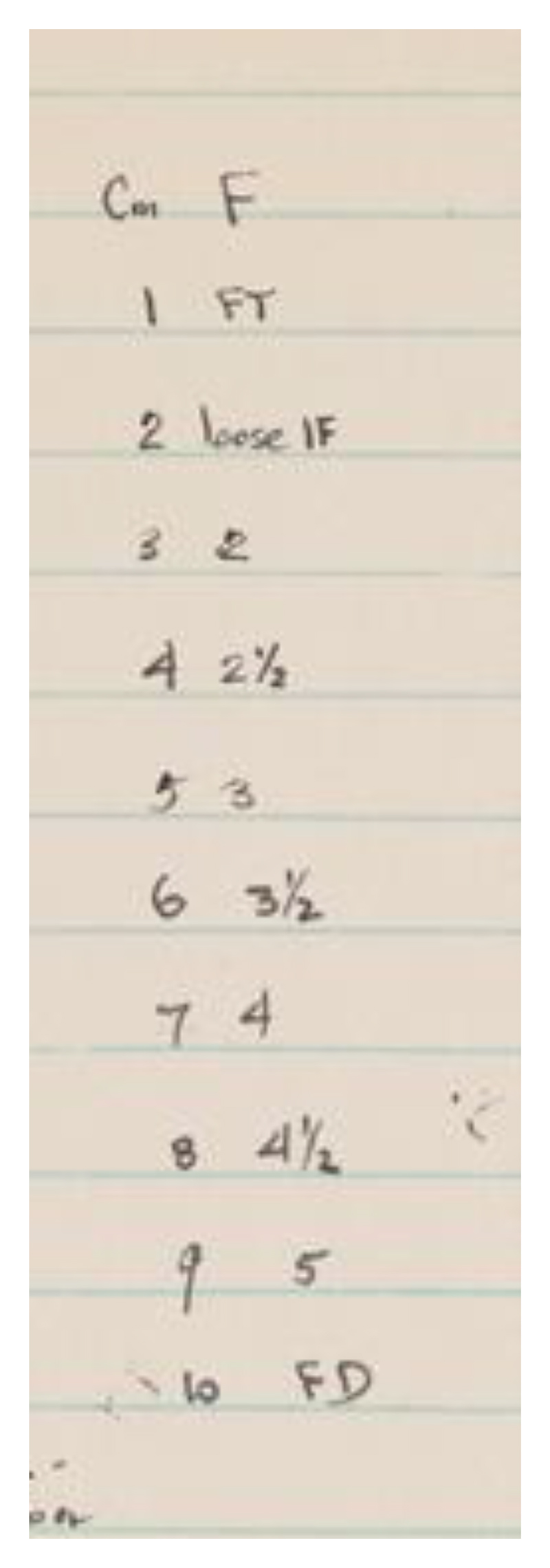
Friedman’s “Fingers to Centimeters” Conversion Chart. Source: Emanuel A. Friedman, “Notebook, Research in the Graphical Analysis of Labor, Sloane Hospital, 1953,” Emanuel A. Friedman Papers, 1953–1989, Archives & Special Collections, Columbia University Health Sciences Library, backmatter.

Rather than intentionally inventing the “one-size-fits-all” tool that would later lead to overdiagnosis of arrested labor, Friedman may even have sought to reduce the number of falsely diagnosed arrested labors, toward the beginning and end of the first stage of labor, due to the comparatively slow rates of dilatation during these phases. Yet the mathematical tools Friedman employed rested on the assumption that the data he was using to build his model were continuous and could be meaningfully placed on an interval scale. Interval-scale data, unlike ordinal-scale data (such as “fingers” and small/large “palm”), can be meaningfully averaged. As someone who paid careful attention to the assumptions underlying statistical tests, he likely knew that this was a weakness of his methodology. He did his best to resolve reliability issues of the digital method (in the sense of “digits,” i.e., by touch) by ensuring that each labor had a unique practitioner estimating dilatation. “All readings therefore became relative,” he observed.^56^ These “relative” readings were considered to be a strength rather than a weakness; each set of patient observations related to a single practitioner’s mental and sensory calibration, eliminating “the variability of the determination of cervical dilatation due to the differences in interpretation” from one measurer to another.^57^ For a study that focused on the changing rate, what was most important was that the units throughout a labor were consistent. He recorded the practitioners’ observations as they reported them, in terms of fingers: fingertip (FT), number of fingers, and full dilation (FD). Then, Friedman created a standard conversion chart in centimeters, likely to ease graphing these points after the fact (see Figure 4).^58^

**Figure 5:**
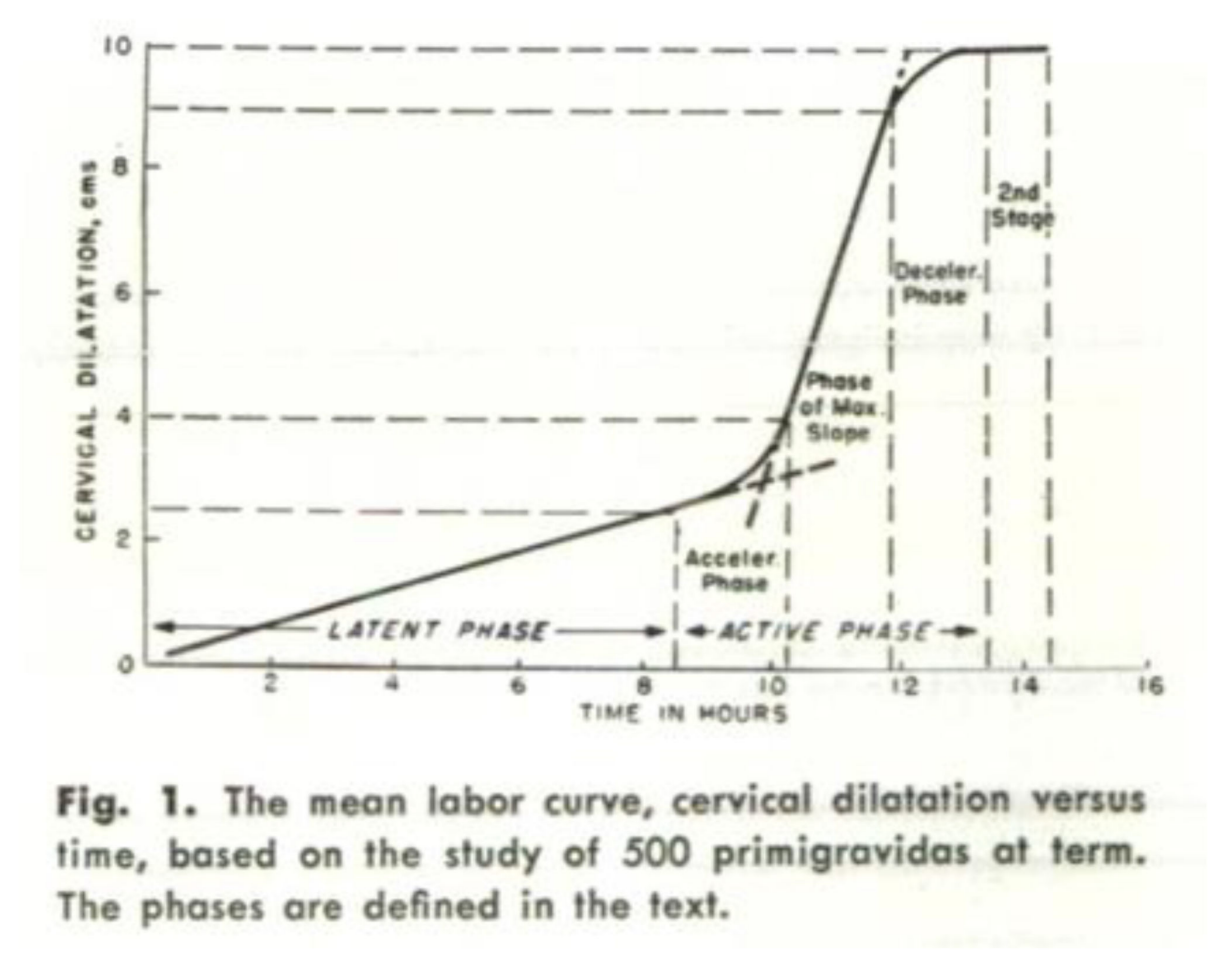
Mean Labor Curve, with Phases of 1st Stage of Labor. Source: Emanuel A. Friedman, “Primigravid Labor: A Graphicostatistical Analysis,” Amer. J. Obstet. Gyn. 6, no. 6 (1955): 567–89.

Because not all hundred women were measured by the same practitioner, the aggregation of these separately calibrated sets of data into summary statistics was merely a convenient assumption. While not particularly warranted by the measuring process, perhaps this assumption was acceptable for an initial study hypothesizing a qualitative trend. In Friedman’s follow-up study on five hundred women published the next year, instead of having an individual labor curve stand as the “ideal,” he constructed a “composite labor curve whose various phases and slopes are those of the means [of the data from five hundred labors]” (see Figure 5).^59^ This new graphically represented composite standard likewise relied upon the assumption that his cervical dilation data in centimeters were more than just a convention or convenience. Without eventually obtaining truly averageable data from which rates could be calculated, Friedman could not convincingly construct his picture of normal labor nor make claims about statistical significance or lack thereof. For example, he could not evidence the claim about the nonsignificance of how long the latent period lasted, regarding eventual labor outcomes. Nor could he convince skeptics that the existence of the deceleration phase was not an artifact of manual measurement.^60^

Friedman needed to obtain further, finer-grained evidence that the sigmoidal curve and the phases of labor that it implied truly represented the normal course of labor by which deviations could be judged. For this to be accomplished, he “awaited the development of a truly objective device whereby the moment-to-moment changes may be accurately measured.”^61^ This “objective device” for measuring cervical dilation, which would eliminate any chance of his curve being an artifact of individual human hands and finally justify the use of interval-scale units, would soon be designed by Friedman himself: he called it the “cervimeter.”

## Friedman’s Mechanical Cervimeter: An “Excellent Academic Tool”

**Figure 6:**
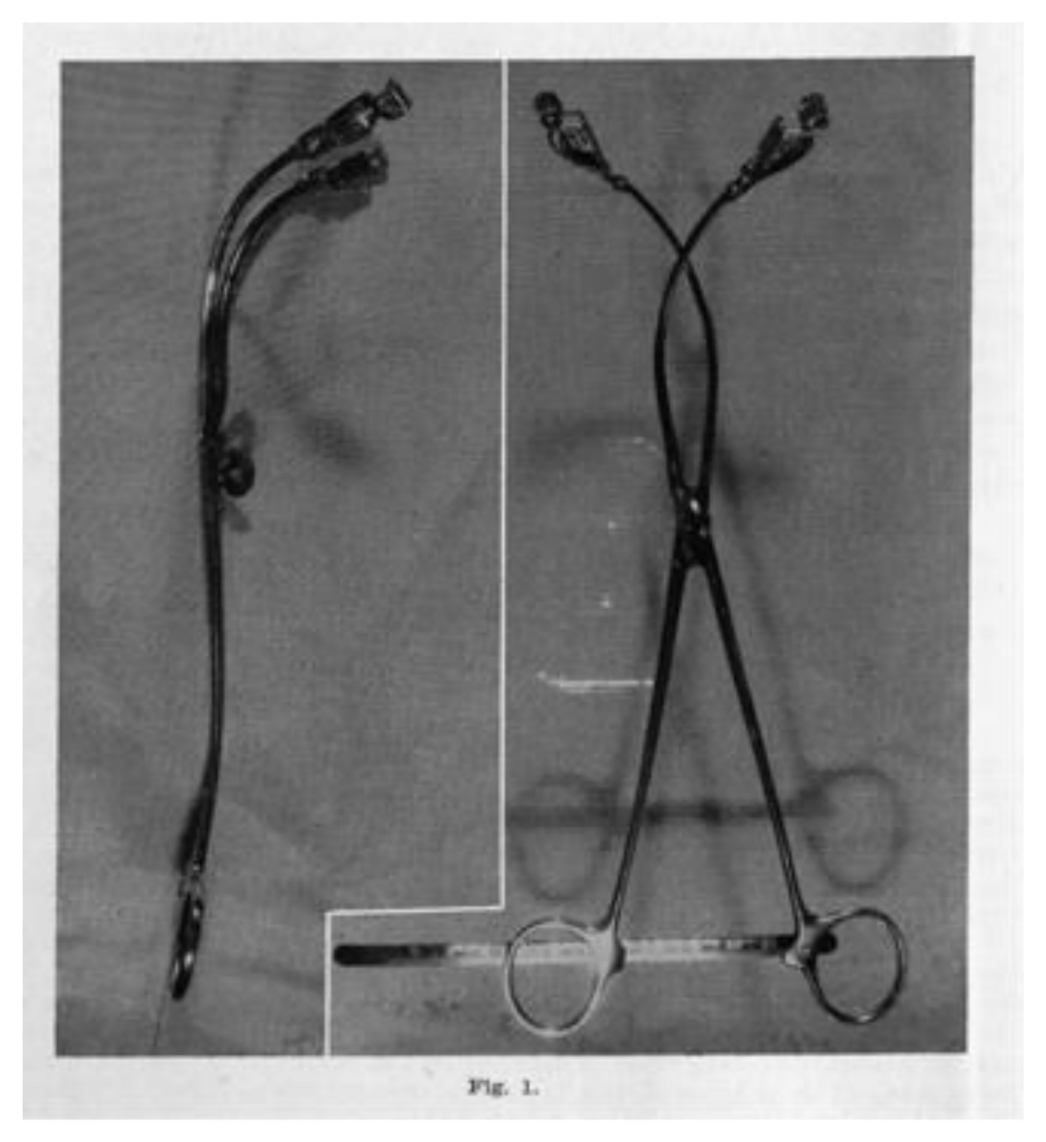
Emanuel Friedman’s Mechanical Cervimeter. Source: Emanuel A. Friedman, “Cervimetry: An Objective Method for the Study of Cervical Dilatation in Labor,” *Amer. J. Obstet. Gyn*. 71, no. 6 (1956): 1189–93.

The tool that Friedman crafted to evidence his sigmoidal curve, and the phases of labor characterized by this shape, underwent several iterations before he reached a satisfying instrument. His first model did not take pelvic curvature into account at all. It was simply two straight “thin nonmalleable metal rods of equal length,” crossed and attached at their centers, with hooks on one end and handles on the other. He wished to take advantage of the simple geometry of similar triangles to avoid the need for calibration: the distance between the hooks was the same as the distance between the handles. But, as Friedman would soon find out, designing an instrument that was theoretically valid for measuring distances on the Euclidean plane did not do much to validate the instrument for use inside the human body. The instrument, as designed, was blocked by “impingement of the levator ani muscles,” which is to say that the patient’s body was getting in the way of the measuring process.^62^ Friedman then curved the hooked ends of the bars toward one another to shorten them, as well as bent the entire instrument laterally to match the pelvic curvature. The result was a modified pair of “25 cm. long uterine dressing forceps,” metal bars articulated at their midpoint with their far ends crossed over, fastened with a thumbscrew.^63^ A triumph of the body’s agency over how it can be measured, the once-straight rods (described as “nonmalleable”!) were nonetheless permanently bent to better conform to the shape of the body. Now, the instrument “no longer measured directly” through the simple symmetry of triangles; instead, the farther apart the handles, the closer the ends were. Because of this, Friedman attached a metal ruler to one of the handles with a hinge, calibrated to reflect the distance between the two ends in *centimeters* (with a maximum reading of 11 cm, as seen on the right-hand side of Figure 6).^64^

Friedman’s early cervimeter models had also experienced problems with attachment, detachment, and harm to the cervical tissue. The hooks were “easily dislodged in the course of labor,” and other more secure methods (such as “suture material” and a variety of “skin clips”) were more difficult to apply and to intentionally detach.^65^ Friedman’s final model featured eight-millimeter serrated bulldog clips to be placed on opposite sides of the cervical opening to hold the instrument firmly in place. To open and close these clips, Friedman used an accompanying instrument, a “Kelly clamp.”^66^ Even this final model was not without attachment problems. The cervical tissue was crushed where the clips were attached, and the process could cause lacerations (an infection risk). One patient’s cervix required suturing to repair a one-centimeter laceration caused by the cervimeter.^67^ Friedman brushed aside these instances; his overall assessment of the impact of the cervimeter on the body was that “no untoward effects were found.”^68^

In addition to physical harm, the instrument imposed constraints on the woman’s birthing position and behavior. Although the shape of metal rods had been curved compared to Friedman’s initial straighter prototype, the final model was still rigid and inflexible. Once the cervimeter was attached, patients were supposed to remain supine during active labor, a severely limiting posture for both laborers and practitioners alike. Yet, the laboring body fought back, continuing to pose problems for Friedman’s cervimeter. One woman, despite attempts to keep her still, sat up abruptly and dislodged both arms of the cervimeter at once.^69^ Another woman dislodged both clips in the course of the second stage (when the fetal head emerges). It is unsurprising that, unlike the hundred women who were measured in Friedman’s original study, the study in which he eventually tested out his “cervimeter” included only twenty-five women.^70^

Regardless of these exceptions, Friedman was satisfied with his final model. The attachment issues were resolved and the shape of the device conformed to the shape of the body it was measuring. Friedman then set about asserting two claims: that his cervimeter was accurate in vivo and that the shape of the dilation curve that he had hypothesized in his 1954 and 1955 studies (an aggregated picture of uncomplicated, first-time labors) was not merely an artifact of manual measurement.

Friedman had already run into problems with assuming the theoretical accuracy of the cervimeter based on design alone. To assert the accuracy of the cervimeter inside the body was a different matter and required a different source of evidence. Friedman claimed that X-ray images verified that the instrument was capable of being properly placed to accurately measure the true cervical dilation.^71^ He then claimed that the readings from the cervimeter were “accurate within 0.5 mm.”^72^ This compared favorably to digital estimations; Friedman stated that digital estimations (from his own unpublished data) had an “average error” of five millimeters, or half a *centimeter*.^73^ His way of assessing “average error” went unexplained, and we do not know what standard he used to compare the two methods to a true distance. It is possible that he made these evaluations outside of the body and then trusted that, because he had shown that the device could be placed in the correct location and stay in place, his assessments of accuracy and error in vivo were well-founded.

Once his cervimeter was shown to be accurate to his satisfaction, his second claim (about the shape of the dilatation curve) could then logically follow. Friedman repeated his previous dilatation study using his cervimeter and obtained similar results, showing that his previous digital assessments were trustworthy and his sigmoidal dilatation-time curve was no artifact. He employed his device throughout the first stage of labor of twenty-five first-time laboring women, recording dilation at two- to ten-minute intervals.^74^ From these data points, he claimed to verify the sigmoidal shape he had hypothesized, this time by “objective” means.^75^ In reporting his results, he emphasized confirming his previous findings both quantitatively and qualitatively.

From the quantitative results Friedman obtained, he was satisfied that his instrument served its purpose in confirming his hypothesized phases of the first stage of labor. Yet he made no claims related to the number of centimeters at which any of these labor phases was likely to occur. The only aggregated data on dilatation in centimeters that he offered was the average maximum slope, a rate of 2.7 per hour (plus or minus 0.40 cm). This figure appears in a list of the average duration in hours of each phase, with an error estimate for each. He did not list average durations for the acceleration or steady phases; he did list that the entire “active phase,” the acceleration, steady, and deceleration phases combined, lasted 6.3 hours on average, plus or minus 1.1 hours.^76^ He seems less interested in the number of centimeters as a meaningful indicator for labor phase than the number of hours in labor, which he acknowledged varied. His evidence pertained to the changes in the rate of dilation and the qualitative information that could be obtained retrospectively by displaying fine-grained dilatation data points graphically.^77^ For example, the question of whether a sedative “slows” the acceleration of dilatation was answered not by referring to specific numerical thresholds but by looking at the changing slope within the acceleration phase (to be discussed in detail below).

**Figure 7:**
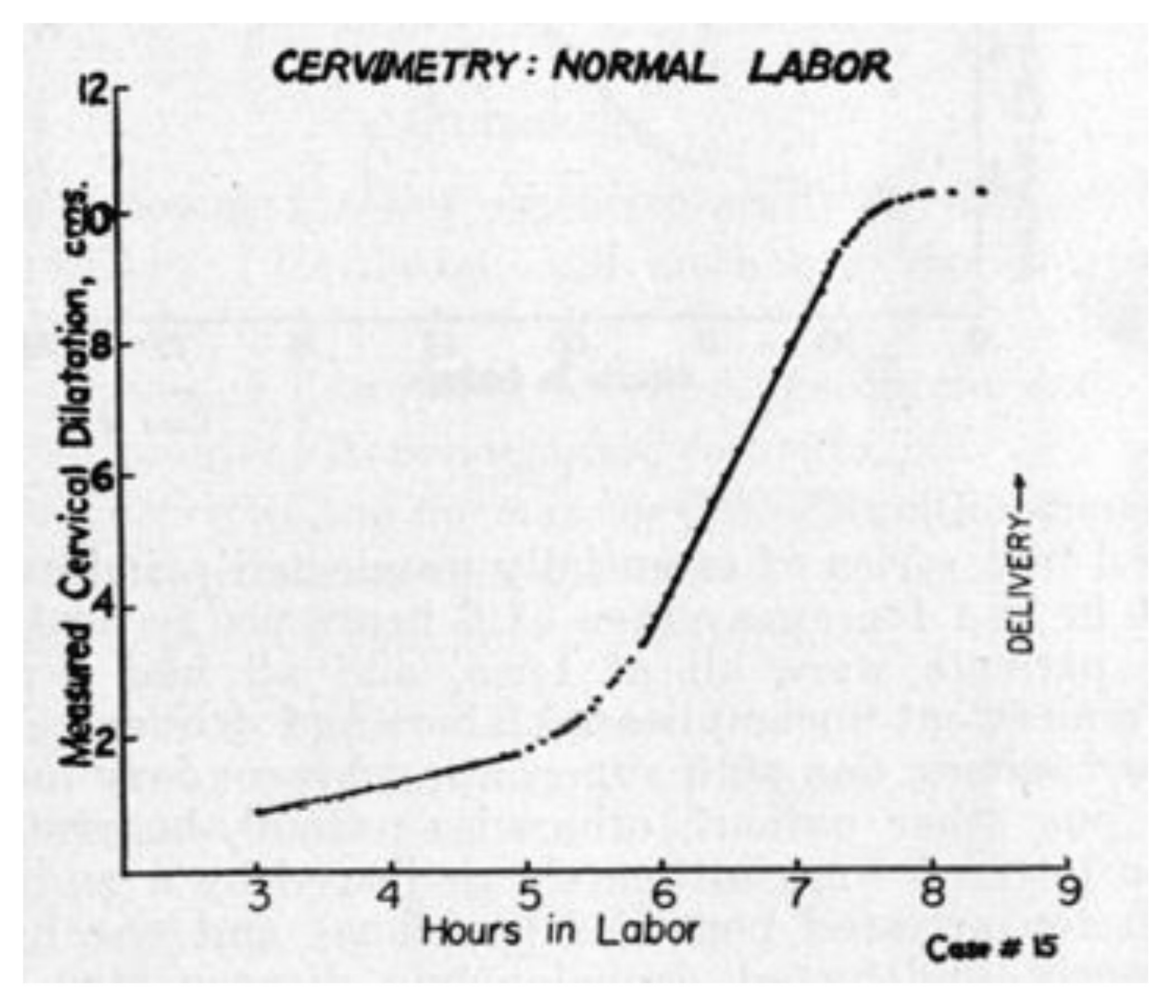
“Normal Labor” Measured by Cervimeter. Source: Emanuel A. Friedman, “Cervimetry: An Objective Method for the Study of Cervical Dilatation in Labor,” *Amer. J. Obstet. Gyn*. 71, no. 6 (1956): 1189–93.

**Figure 8:**
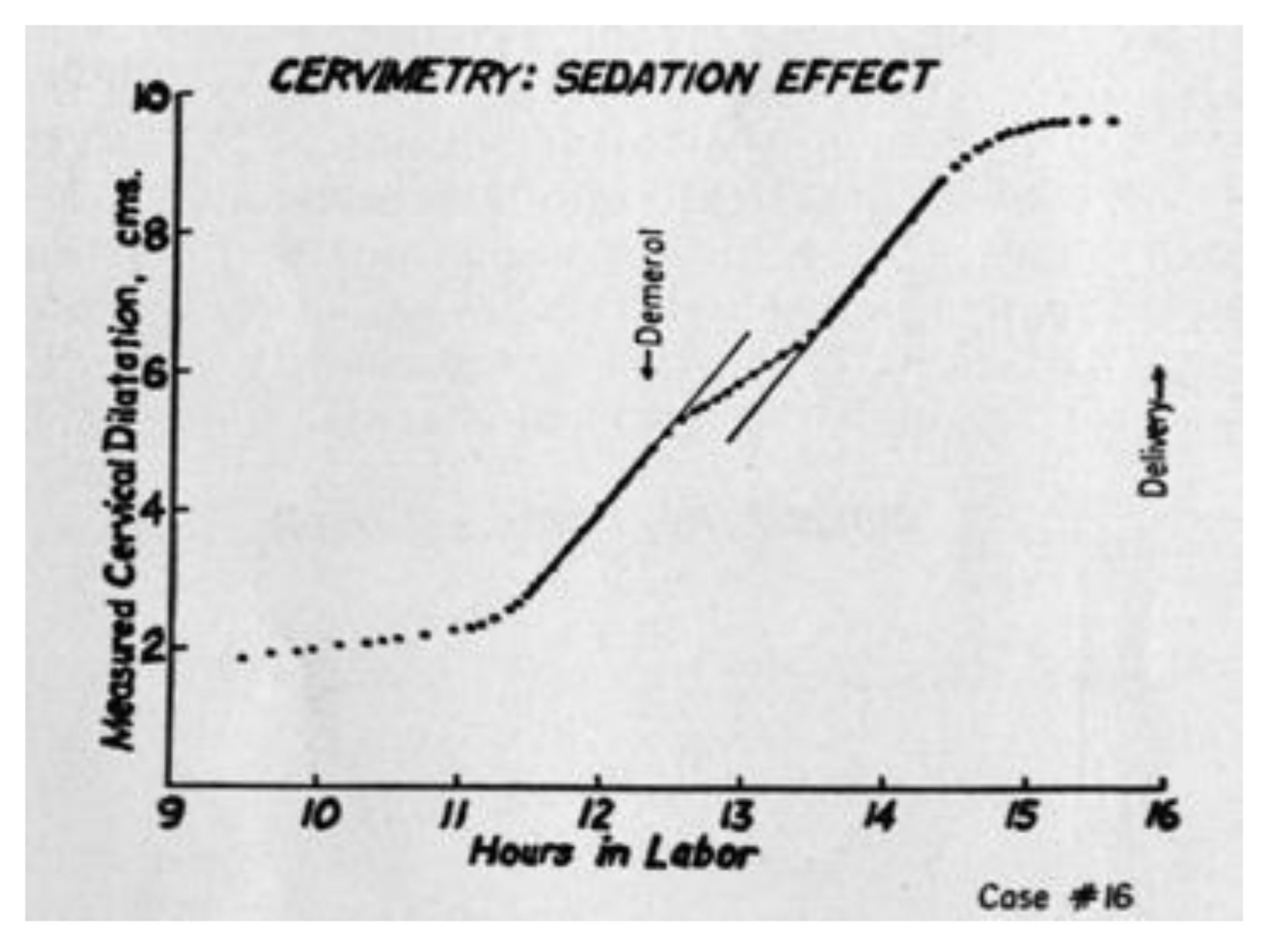
Sedated Labor Measured by Cervimeter. Source: Emanuel A. Friedman, “Cervimetry: An Objective Method for the Study of Cervical Dilatation in Labor,” *Amer. J. Obstet. Gyn*. 71, no. 6 (1956): 1189–93.

Qualitatively, he confirmed the slow growth of the “latent phase,” before the dramatic increase in dilatation rate (the “acceleration phase”). He also confirmed the slowing growth of the “deceleration phase,” which he considered to be the more controversial of his claims. According to Friedman, the dilatation rate flattened out just before arriving at full dilation. This deceleration was doubted by Leroy A. Calkins (chair of obstetrics and gynecology at University of Kansas), according to Friedman’s personal communications.^78^ A single curve was selected to represent the “normal” and “abnormal” cases of deceleration, respectively. The graph of “Case 15” (Figure 7) displays the prototypical sigmoidal pattern that Friedman claimed to have observed occurring in all twenty-one of the “normal uncomplicated labors.”^79^ Yet Friedman pointed out that even in these ideal, best-case scenarios, there was no small variation in labor duration and dilatation rate. This did not seem to surprise or concern Friedman. What these “normal” labors shared, and what Friedman was most concerned about, was their overall *shape*. Accordingly, “Case 16” (Figure 8) is displayed as the prototypical example of errancy from the expected S-shaped curve. The deceleration (flattening of slope) occurred prematurely—that is, during the acceleration phase. This is evident by virtue of the fact that the dilatation picked back up to its former rate (at about 13.5 hours) before the eventual expected denouement approaching 10 cm. Friedman attributed this event to “large doses of analgesic-sedative medication” and pointed out that this subtle shift would easily have been missed if less-frequent data points had been collected.^80^ He saw his cervimeter as providing as-close-to-continuous-as-possible data, for investigating and comparing the effects of drugs and other interventions on the course of labor.^81^ The infrequent and irregular data collected by digital examination were liable to miss such qualitative subtleties.

It is worth reiterating that Friedman’s cervimeter, for all its hard work at obtaining numerical precision, was also not particularly intended to be used for discrete representation of cervical dilatation at any given point but rather was meant to offer a finer-grained representation of the changing rate of dilatation. This is apparent not only from his own claims about what he sought to achieve but also from the design of his graphs (see Figures 7 and 8). It is difficult to tell the exact number of centimeters that any given point represents. Instead, the qualitative features of the sigmoidal curve are what is readily apparent: the gently sloped latent phase, the steep acceleration phase, and the flattened deceleration phase. Friedman made little effort to make his graphed points individually legible and did not list any thresholds in centimeters for when a given phase typically occurs. His emphasis on rate, and the qualitative attributes of the phases of labor, was clearly inspired by the original question that prompted his inquiry: How can we ascertain whether a given intervention qualitatively affects the labor process, in a way that respects the normal variations of individual labors?

Interestingly, it seems that Friedman did not consider his cervimeter fully validated before using it in his study, but rather it was validated by the data it produced in the study. He compared the dilation data as estimated by his cervimeter, namely the duration of labor phases and maximum dilatation slope, with similar figures from previous studies that used digital estimation. Indeed, digital methods, being the only other methods for comparison, were used as an evidential backing for validating both his cervimeter and the sigmoidal data it produced. Despite this, he subsequently referred to digital methods as “far less accurate determinations of dilatation” and claimed that his cervimeter’s data in turn actually validated the use of digital methods.^82^ His cervimeter data indicated that “the error associated with rectal and vaginal examinations is relatively small when compared with the wide variations among labors,” and thus “the alleged inaccuracy of digital examinations in labor, upon which the original studies [which showed the sigmoidal pattern] were based, has been disproved by inference.”^83^

While this logic may seem circular, recall that the primary reason Friedman wished to validate his new device was to rescue his previous studies from the criticism that his results could be biased by digital measurement. The reason to mistrust the results of digital assessments was the nonuniformity of its units. In contrast, the reason to mistrust the cervimeter was the possibility of in vivo inaccuracy, such as the instrument slipping off its target or being placed incorrectly in any given instance. These two methods, with nonoverlapping weaknesses, agreed in their aggregated estimates and arrived at the same qualitative patterns. The complementary nature of the flaws of these two methods allowed for them to act as mutually evidential;^84^ the qualitative results they each produced were less likely to be mere artifacts of instruments with such very different flaws. With this in mind, mutual agreement between the instrumental and manual methods was validation *of the sigmoidal curve*, Friedman’s foremost concern.

Friedman concluded that the cervimeter was a success, an “excellent academic tool” for experimental research. That said, he noted that “frequent digital examination is a much more expedient” method for clinical use.^85^ He did not expect nor intend for his device to ever be adapted for such uses in the future, as “the variation and duration among labors is far greater than the superior accuracy of the cervimeter warrants,” and thus there was no sensible reason to do so.^86^ Friedman had groups of labors in mind as the relevant unit of analysis for this measuring instrument, as this was the scope of measuring that would be relevant for testing interventions in the research context.

Seven years later, Friedman partnered with Lajos I. von Micsky in a second attempt at measuring dilation continuously. Friedman and von Micsky hoped that tracking dilation patterns continuously and graphically, in tandem with uterine contractions, would help with understanding the mysterious relationship between the two. In this interest, they created an electromechanical cervimeter with flexible cables (allowing for more birthing positions), which sent electronic signals to the mechanical arm of a recorder. This recorder drew continuously on a ream of paper, so that one could see continuous measurements in real time and preserve this record for future analysis. Sadly, the flexibility of this new instrument, while reducing undue influence on the laboring body, had the trade-off of allowing more influence from the body on the instrument. They discovered that the cervix responded to the device (attached by metal needles that pierced the cervix on opposite sides in order to stay in place) by dilating asymmetrically. Past 7 cm of dilation, the device was pushed so far forward (closer to ten o’clock and two o’clock) that it no longer meaningfully recorded the dilation diameter as intended.^87^ Yet again we see the agency of the laboring body, pushing back (literally) against standardized measurement.

After this failure, Friedman never returned to the project of making a cervimeter.^88^ Even thirty years later he responded to another group’s attempt to make such a device, warning that any cervimeter that had sensors attached to opposite sides of the cervix would face difficulties detecting the deceleration phase, due to the cervix’s ability to dilate asymmetrically and distort the measurements.^89^ Friedman put to bed his project of creating a continuous measuring instrument (even for purely research purposes) and had always rejected the notion of a cervimeter having any role in clinical labor management.^90^ Yet this was “wisdom that is too soon forgotten”; Friedman’s cervimeters inaugurated decades of (failed) attempts at creating a reliable cervimeter for clinical use, throughout the twentieth century and still today.^91^

Much like how the original context and intentions behind Friedman’s cervimeter were quickly lost and a standardized apparatus was later pursued for its own sake, the use of “centimeters” of dilation had a similar trajectory, but with greater lasting impact. Measuring *centimeters* of dilation over time, accompanied by the partogram as a central tool, fundamentally shaped labor management in the latter half of the twentieth century—but not in the way that Friedman had originally intended.

## The Legacy of Friedman’s Curve and Cervimeter: Interval-Scale Thresholds and the Auditor’s Pen

**Figure 9:**
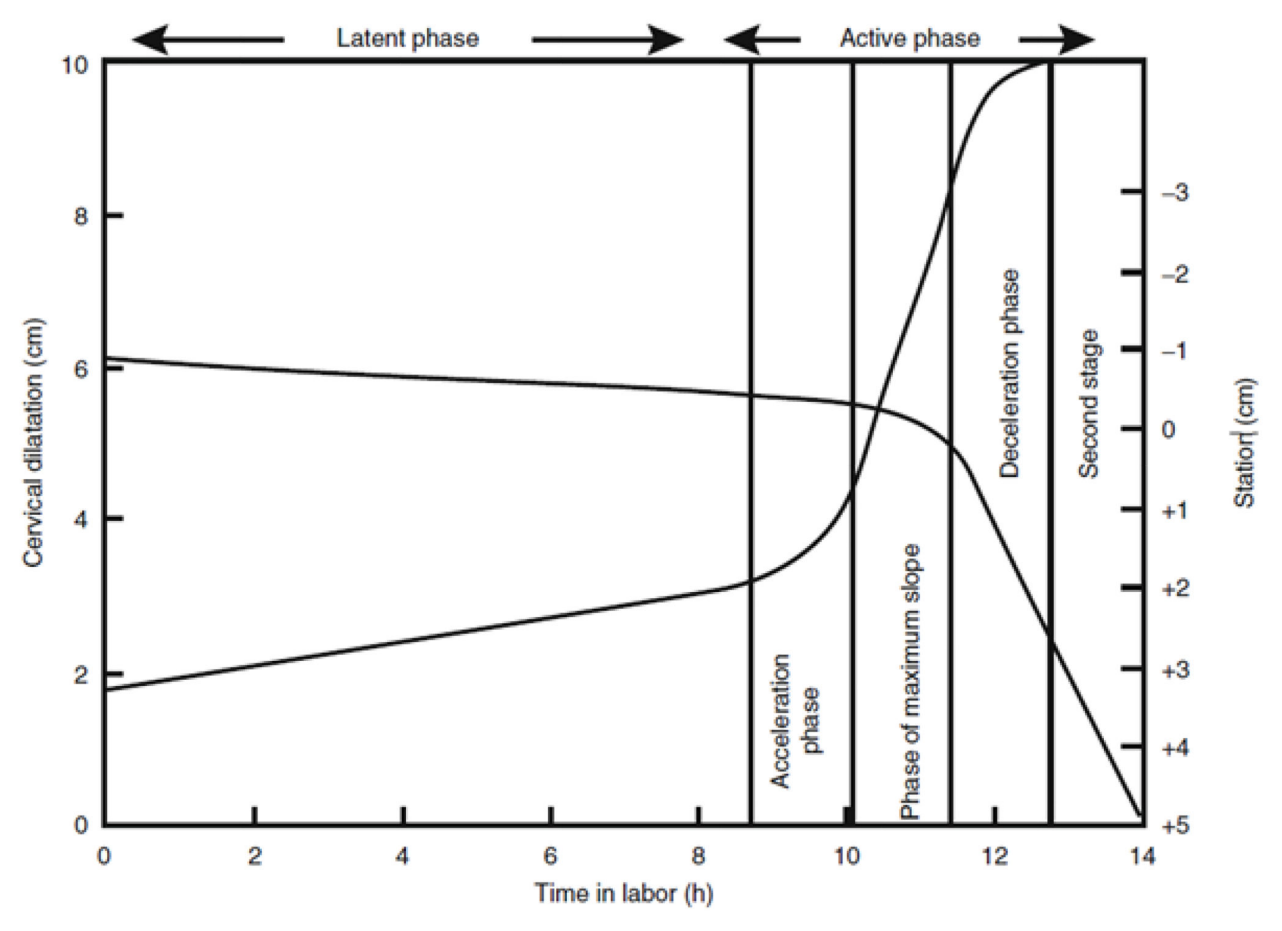
The Friedman Curve. Source: Ariel L. Zimerman, “The Use of Two-Dimensional (2D) and Three-Dimensional (3D) Ultrasound in the First Stage of Labor,” in *Intrapartum Ultrasonography for Labor Management*, ed. Antonio Malvasi (Berlin: Springer, 2012), 29–40, 30.)

**Figure 10:**
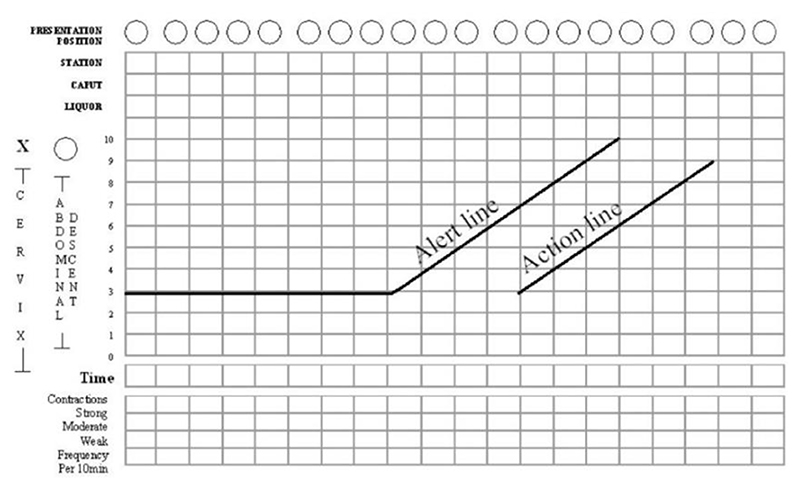
Example of a Partogram Template. Source: Tina Lavender, Anna Hart, and Rebecca Smyth, “Effect of Partogram Use on Outcomes for Women in Spontaneous Labour at Term,” *Cochrane Database Syst. Rev*. 10, no. 7 (2013): CD005461.

**Figure 11:**
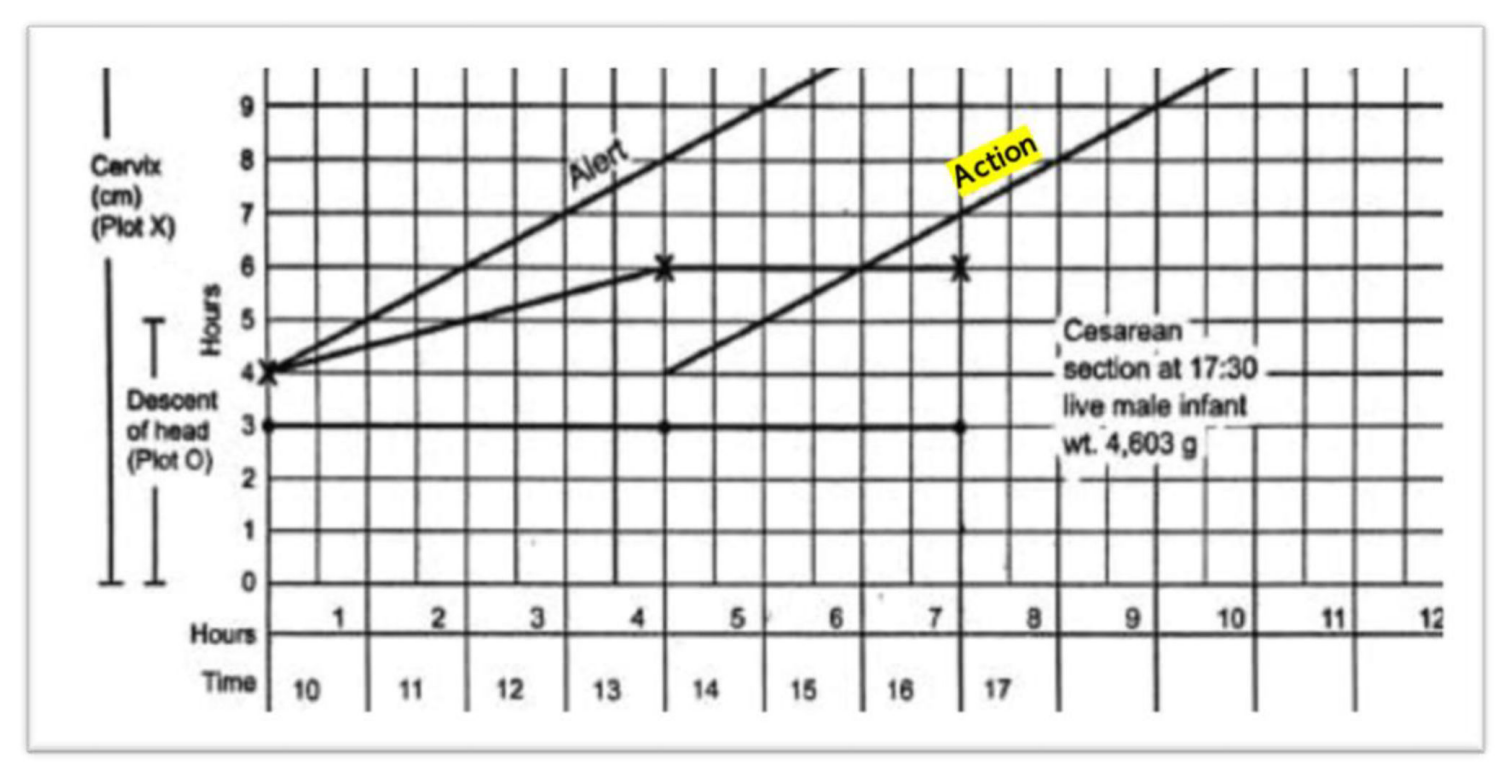
Partogram of Labor Resulting in Cesarean Section. Source: Asha R. Dalal and Ameya C. Purandare, “The Partograph in Childbirth: An Absolute Essentiality or a Mere Exercise?,” *J. Obstet. Gyn. India* 68, no. 1 (2017): 3–14 (shading and typo correction mine).

What began as a summary of data, from which Friedman was able to make qualitative observations he hoped would prove useful for evaluating each labor on its own terms, was eventually transformed into a standard for establishing numerical dilatation thresholds for hospital policies and interventions. One can easily come across Friedman’s sigmoidal curve in textbooks on obstetric standards, including one that appears in a chapter on the merits of 3D ultrasound technology (Figure 9). Here, the dilatation curve is reproduced as a guide for normal first-stage labor (mapped alongside a downward curve representing the baby’s station). The phases of labor have become canonized to begin at certain dilation thresholds, and accordingly, accurate cervimetry assessments constitute “the most critical parameter in the management of the first stage of labor.”^92^ This is a natural conclusion one would reach about labor according to the diagram of labor progress, now called a “partogram” or “partograph,” which the author considered “the central pillar for clinical management of labor today.”^93^ Most hospitals and clinics in wealthy countries use a partogram for each labor;^94^ the partogram allows for graphing cervical data over time, with templated “action lines” that signal when a safe dilation level threshold has been crossed and intervention is necessary (Figures 10 and 11).^95^

**Figure 12:**
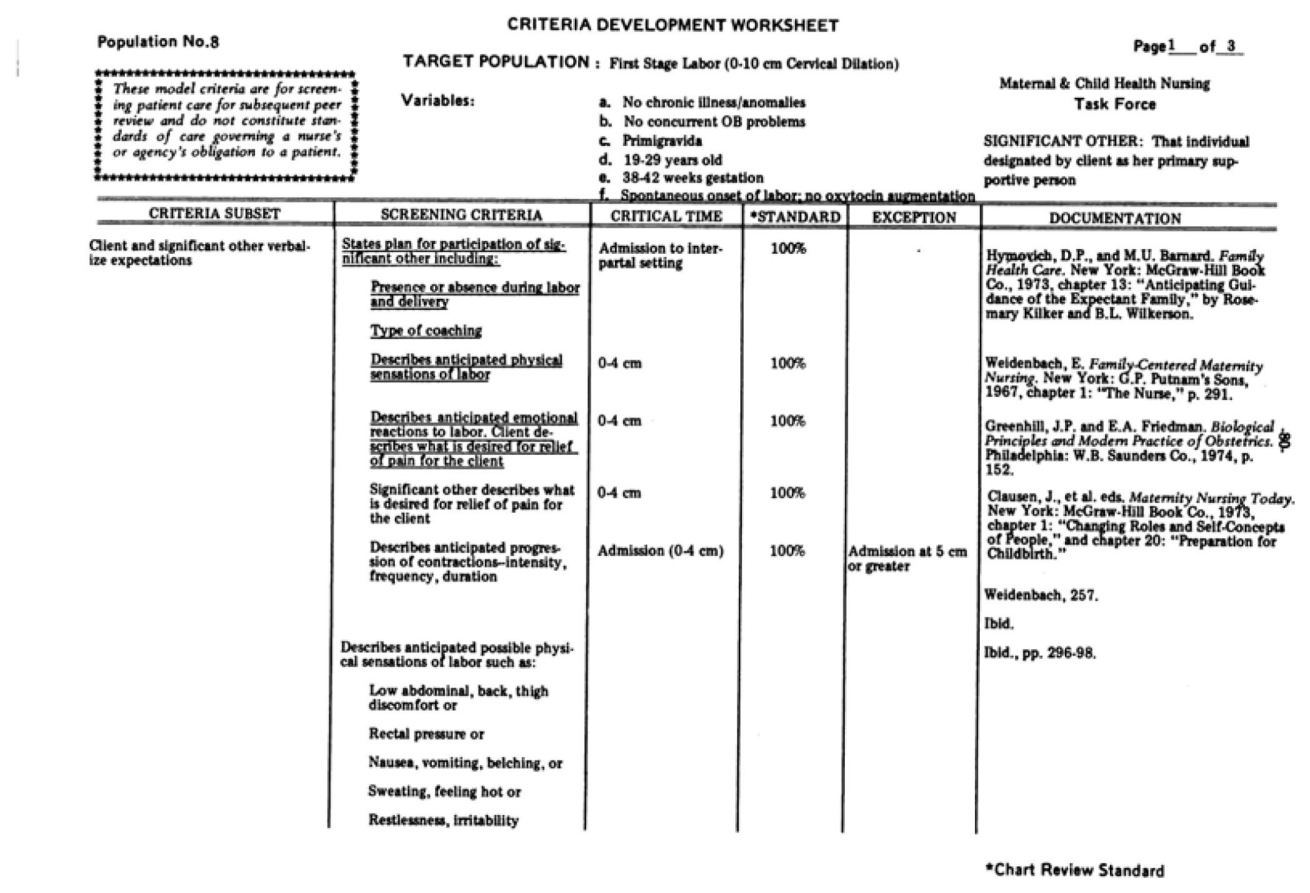
First Stage Labor Patient Care Screening Worksheet Page 1. Source: American Nurses Association, Health Services Administration, Bureau of Quality Assurance, “Guidelines for Review of Nursing Care at the Local Level: Emphasis Given to Professional Standards Review Organizations and the Use of Outcome Criteria in the Review of Nursing Care” (Kansas City, Mo.: ANA, 1976), 80–82.

**Figure 13:**
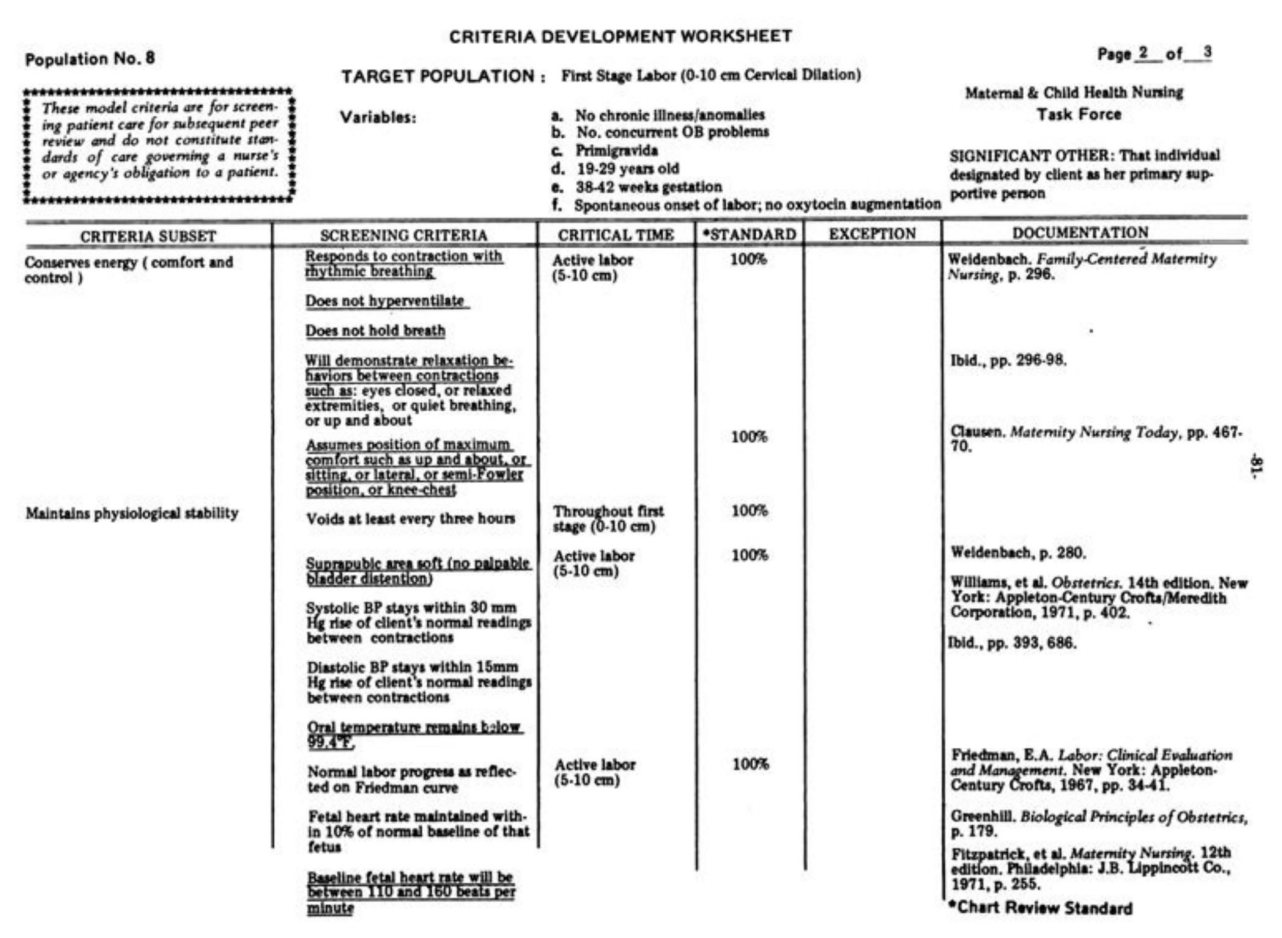
First Stage Labor Patient Care Screening Worksheet Page 2. Source: American Nurses Association, Health Services Administration, Bureau of Quality Assurance, “Guidelines for Review of Nursing Care at the Local Level: Emphasis Given to Professional Standards Review Organizations and the Use of Outcome Criteria in the Review of Nursing Care” (Kansas City, Mo.: ANA, 1976), 80–82.

Tracing how exactly Friedman’s dilatation-time curve impacted clinical standards and practices—particularly, how the dilatation-time curve became institutionalized in the use of the partogram—is a subject worthy of further historical inquiry. While a complete answer is beyond the scope of this article, we can get a hint of how this transformation may have occurred by looking at a document from the 1976 “Guidelines for Review of Nursing Care at the Local Level,” coauthored by the American Nurses Association, Health Services Administration, Bureau of Quality Assurance in the United States.^96^ The worksheet in Figure 12 was used to audit nursing care records according to best practices, as defined by “critical time” listed in centimeters of dilation, and justified by white papers (listed in the rightmost column), among which Friedman appears twice. One screening criterion in particular lists explicitly, “Normal labor progress as reflected on the Friedman curve,” during active labor (defined as 5–10 cm) (Figure 13). While the audit worksheet emphasizes that these standards are “for screening patient care for subsequent review” and “do not constitute standards of care governing a nurse’s or agency’s obligation to a patient” (see Figure 12), it seems difficult to believe that auditory standards would not in turn affect standards of care over time.^97^ Consider that, just four years prior, the American Hospital Association passed their first Patient’s Bill of Rights (1972) which expanded patient rights and protections to include the right to refuse treatment and access to information about their past and current treatments. This was just the beginning of an era that reshaped the previously one-way relationship between patients and their medical data.^98^ By the 1980s, “informed consent” was solidified as a legal term that could be used to force transparency between what doctors know and what patients decide. Patients’ increased access to their own records may have contributed to the shift in labor measurement practices toward meeting quantifiable standards. Individual clinical data and related care decisions needed to be recorded and preserved *for patients* (and their legal representatives) to reference for their needs and purposes, not just for physicians and researchers.

On the nursing care audit sheets, qualitative and quantitative features of labor are—in a checklist fashion—based on the centimeters of dilatation (interpreted as the stage of labor). This is a far cry from cervical dilatation trends as being helpful and numerically recordable indicators, alongside others, as Friedman saw them: “Cervical-dilatation-time relationships, expressed graphically, yield considerable information regarding progress of labor.”^99^ Cervical dilation was not always thought of as a proxy for labor progress. Yet the measurable part of labor became increasingly conflated with the labor itself. Friedman’s distaste for defining normal labor based on thresholds of dilation states rather than changes in dilatation rates is still recognizable today. In a recent interview, he complained that “the acceptance of 4 cm as the onset of the active phase is puzzling.… The woman herself, the laboring patient, describes her own labor curve. From my perspective, it’s inappropriate to arbitrarily designate any given point in the cervical dilatation to the onset of … the active phase.”^100^

Until a very recent push to change the guidelines for labor assessment, the Friedman curve has been (mis)used as the primary standard for a “normal” length and rate of labor and dilatation. Despite Friedman’s own intentions to provide an individualized standard for labor care, the definitions of “normal” and “abnormal” labor have remained generalized and based on predetermined thresholds. By this standard, first-time mothers are allowed about fourteen hours to advance to 10 cm, and experienced mothers are allowed only eight hours. In 2014, ACOG and SMFM determined a new, more flexible set of guidelines, intended to prevent unnecessary cesarean sections in response to “arrested” labor.^101^ Considering the epistemic humility Friedman showed in his early work (which acknowledged that even perfectly healthy labors varied widely in length and offered only general windows for the lengths of labor phases), one might suppose that Friedman would be uncomfortable that his name is attached to a standard that has led to nearly one in three laboring women undergoing cesarean sections in the United States.^102^ Yet Friedman responded to the new 2014 guidelines with deep skepticism, saying that they were “misguided” and risked the health of women by employing standards that have not been as empirically vetted as his Friedman curve.^103^

Such evidence is now in the process of accumulation. A 2016 prospective study of 419 Italian first-time laborers was conducted, wherein half received the old model of care that adhered to the Friedman’s curve guidelines and the other half received the new model of care, similar to that proposed by ACOG/SMFM. The new guidelines, rather than using strict thresholds for determining courses of action, considered arrested or protracted labor as simply being a “warning sign promoting further diagnostic assessment prior to considering intervention.”^104^ Within this new perspective on labor decision-making, “cervical dilatation curves are ‘downgraded’ to the category of a screening test” for abnormal conditions, among several other indicators used to diagnose issues, in an effort to avoid any significant augmentation of the labor process.^105^ The study concluded that women who received the new model of care had half the rate of cesarean operations as compared to those with the old model of care, which adhered strictly to Friedman’s curve (10.3 percent vs. 22.2 percent). These women also benefited from the new model by having fewer overall interventions, such as Pitocin to stimulate contractions or having their water artificially broken.^106^

We may wonder why this movement toward more nuanced birthing guidelines is not accompanied by a change in the “centimeter” notation for recording digital cervix assessments. If the central role of Friedman’s curve in obstetric care weakens, there would seem less warrant for interval-scale dilatation data to be recorded. Additionally, evidence continues to accumulate that should lead us to question whether cervical exams recorded in centimeters are as medically informative as we treat them. Helen Feltovich pointed out that “even the need for intrapartum cervical evaluation is debatable; a Cochrane review of intrapartum vaginal examination reported that knowing dilatation does not help predict timing of delivery” and cited a study conducted by midwives that concluded that “cervical examination is uncomfortable, uninformative, and, ultimately, unnecessary” and that less-invasive alternatives exist.^107^ Yet there may be reasons, beyond simple institutional inertia, to believe that both vaginal exams and the language of “centimeters” in describing dilatation will indeed persist. One reason relates to why there is resistance from the legal community to the newly proposed flexible obstetric standards.

Although from a patient-care perspective it appears that it is time to retire the Friedman curve, medical malpractice lawyers are not so sure. Without a way to establish “normal” labor and “normal” obstetric decisions, it is more difficult to litigate malpractice suits and prove practitioner negligence or unwarranted risky interventions. One law firm that focuses exclusively on birth injury cases, American Baby and Child Law Centers (henceforth ABC Law), posted a statement on their website in 2017 titled “Rewriting the Rules of ‘Normal Labor’: What Does This Mean for Mothers?”^108^ This statement heavily critiqued the proposed shift from the Friedman standard.^109^ Rather than seeing flexible guidelines as a patient-centric approach to labor management, ABC Law denigrated the 2014 recommendations by the ACOG and SMFM as a “‘wait and see’ approach” that “does a grave disservice to mothers and children by exposing them to greater risk to injury.”^110^ In comparison, they noted the advantages of the Friedman curve: “The *Friedman curve was a simple and effective* method for *helping doctors* make clinical recommendations, allowing them to *recognize and quantify* the effects of parity, analgesia, obesity, prior C-section effects, maternal age, fetal position and presentation over 60 years—things the new, unvalidated methods could not do.”^111^ In short, the Friedman curve offers malpractice lawyers quantifiable standards of evidence based on large numbers of laboring women in research studies. The advantages cited above, namely the simple and clear standards for quantifying “effects” of various aspects of labor, relate to the collection and evaluation of aggregated data of *other* women in retrospect, not with guiding the safe labor of an individual mother or child in the moment of delivery. This would appeal particularly to those who will be arguing for or against the reasonable behavior of the doctor; the rhetorical advantage of a general standard does not necessarily advantage the woman who is actively in labor. If the Friedman curve were to be set aside, the ABC Law lawyers would be forced to look to other quantifiable data to argue their cases; resorting to other data, such as fetal blood gas levels,^112^ would allow for fewer labors to be considered abnormal or stalled. Likely, the patient-clients of these firms would feel similarly resistant. Even if relying on dilation data in *centimeters* is not a clinically meaningful way to guide, standardize, or audit labor management practices, from a patient perspective it could seem like a loss to give up such a significant source of seemingly “hard” evidence that their care was not well managed.

The legal concerns surrounding obstetric measures resemble what Ted Porter has argued occurred more broadly in twentieth-century U.S. science, noting that “courts have been particularly stubborn in believing that science should mean the straightforward application of general laws to particular circumstances” to the extent that by the 1980s the testimony of real living experts “often holds up rather badly” in comparison.^113^ The prioritization of seemingly objective (though empirically ineffective) standard units allows for a “withdrawal of human agency” on the part of the medical practitioner, who has only to follow the Friedman standard to avoid liability; as Porter has noted, “subjectivity creates responsibility.”^114^ As obstetric medicine searched for general standards and strove to construct scientifically “objective” forms of measurement, it also turned toward bureaucratic strategies of management and recordkeeping. Obstetric units of measurement, accordingly, are bound up in this system of hospital liability protections, a central piece in fulfilling the obligation to render clinical observation recordable and clinical judgment legible to broader audiences after the fact.

While the “Friedman” standard may be the most common reason for dystocia diagnoses treated by cesarean section, it is far from the only reason for the increased cesarean rate in the United States and beyond.^115^ Other obstetric technologies such as the Apgar score, the Bishop score, ultrasound imaging, and electronic fetal heart monitoring have played a role in promoting certain visions of risk, enforcing or altering professional boundaries to disadvantage midwives and incentivize hospital practitioners to err on the side of defensible actions even against their own better judgment.^116^ Yet the role of measuring units themselves as rhetorical devices that can be used in favor of defensive medicine after a birth (or, in contrast, understanding which units can best serve patient safety) deserves more attention. Future historical work exploring the relationship between cervical data and practitioner liability should examine the argumentative strategies employed in obstetric malpractice suits (by both the prosecution and the defense). If data from partograms, particularly dilation in *centimeters*, are referenced heavily in these arguments, this could point to an area of tension in obstetric measurement best practices. As Dan Bouk has argued, paper technologies, rather than being immutable records that are transported without changing meaning or use, are “useful precisely because of the multiple purposes they could serve as they moved not only through space but through time.”^117^ Just as the partogram had the potential to transform from primarily a clinical guide in the moment of labor to serving a dual role as a legal record for protecting hospitals from future litigation, so the unit “centimeters” seems better poised to serve purposes after the fact. Units of measurement, whether embodied as objects or as symbols preserved through objects, share this potential to be mutable, mobile, and multipurpose records across time-space contexts. With this flexibility comes the threat of conflicts and collisions when units must be designed to time (and space) travel for use and interpretation by multiple audiences with different priorities. The demands we have for measured information in the high-stakes moment of clinical decisions may in fact be at odds with the potential legal demands we may have from this same information in the future.

## Conclusion: How Constructing *Centimeters* Reconstructed Labor

Instead of measuring labor progress by the increase in regular labor pains or estimating danger by the length of the labor, this story shows how cervical dilation in *centimeters* takes on the role of proxy measure for representing both labor progress and risk. It was not an inevitability that cervical dilation would become this measure. The role of the cervix in the early twentieth century was one of a physical barrier, and dilation was regarded as a corporeal limit to be observed and respected. Unnecessary cervical tears were thought to result from an unskillful and impatient attendant and were a mark on one’s professional reputation as a physician. By the mid-twentieth century, if any single variable seemed to be acting as the replacement for the information previously gained from attending to the woman’s labor pains, it was the sheer number of hours in labor.

It was also not an inevitability that cervical dilation would ever be measured with a truly interval-scale unit (*centimeters*), which could then be used in setting hospital maternity care standards (and in the American context, arguing legal cases). As discussed in the second section, the role of recorded numerical cervical dilation in mid-twentieth-century Europe was in evidencing general research claims;^118^ yet, even for this use, it was criticized as too broad of a brush to use for arriving at clear conclusions.^119^ When recording dilation data, obstetric researchers were just as likely to use a nonuniform, ordinal unit like “fingers” in such studies. Even when “centimeters” were nominally used in studies, this was likely a translation from units derived from familiar handheld objects (such as coins, watches, and wedding rings). These objects were used for training practitioners to make simple ordinal comparisons (aiding judgments of changes such as “greater, lesser, and similar”); thus, it is not too surprising the objects in the scale varied in their differences between one another, and the object-units themselves were translated into centimeters only by loosely approximated “averages.” We can easily imagine an alternative historical path in which practitioners settled on an ordinal unit system for dilation, similar to how cervical consistency/softness is measured today on a scale of “soft, medium, and firm.”^120^ We could imagine a system of units for cervical dilation that consisted of, for example, “closed, small opening, mid-opening, nearly full, full dilation.”

Today’s reality of recording dilation in *centimeters*, treated as a scale for labor progress, required active construction. And yet it was also an accidental by-product of constructing the dilatation-time function (now known as the Friedman curve) and the cervimeter. While both the curve and the cervimeter were American inventions that had global impacts on labor management standards, the historical role of the latter has been overlooked. The third section showed that Friedman’s expectation was that the *shape* of his dilatation-time function, an S-shaped curve described by changes in centimeters-per-hour, would become the relevant guiding principle by which obstetricians would make judgments in real time. To support his claim about the sigmoidal curve, as the fourth section explained, he needed an instrument to verify that the shape of the curve (the slope) for uncomplicated labors was a reality, not an artifact of manual measurement. To argue convincingly about subtle changes in centimeters-per-hour, he needed his audience to be able to take the “centimeters” part seriously. Only once *centimeters* of dilation were constructed was it possible for standards based on dilation (as a truly interval-scale phenomenon) to take on a life of their own, establishing thresholds for defining labor activity and dictating when intervention was necessary. This transformation was aided by the use of two hospital paper technologies, partograms and auditing checklists, as briefly discussed in the previous section.

Emanuel Friedman’s original intention was to individualize labor guidelines and refocus practitioners on the dynamic body itself. Instead, the abstraction of labor down to a single dimension is the (perhaps unfortunate) legacy of Friedman’s work. To arrive at the partogram and the centimeter-based labor standards we have today required two pieces of technology: the Friedman curve and the cervimeter. From these, two steps were achieved: the reification of dilatation as a proxy for labor progress and the reification of dilation as an interval-scale phenomenon (in *centimeters*). Together, they gave us *centimeters* of dilation from 0–10 as the scale for labor progress itself.

## Epilogue: Dismantling *Centimeters*

Dilation may be a poor heuristic, as it turns out, to centralize labor decisions around. Yet we have certainly seen worse.^121^ The unit of “centimeters-per-hour,” as Emanuel Friedman thought of his contribution, pointed the birthing attendant to some aspects of the birthing body and away from others. While the holistic reality of birth as a physiological and psychological process was missed in this transition, some of what was lost may have needed to be discarded. Though Friedman kept track of pelvis type, age, and perceived race of the women subjected to his experiment with the raw results in his notebook,^122^ this information is scrubbed from the results of his published studies and thus deemphasized in its relevance to predict birthing outcomes. The unit of “centimeters-per-hour,” and even “centimeters,” obscures the differences between birthing bodies that turned out to be less clinically significant than they were thought to be in the nineteenth century (and much of the twentieth). Although returning to recording dilation in “finger” units again may very well be an improvement, our response to knowing this history should not be a nostalgia for the obstetric practices of the 1950s United States. As always with history of measurement in medicine, we can appreciate the best accidental results of constructed standards, and any social and epistemic gains along the way, while discarding uninformative and harmful instruments from use. Friedman employed his cervimeter for a research purpose, found it flawed for any clinical purpose, and discarded it. This history should make us question whether *centimeters* of dilation, like the cervimeter, belong on a shelf in a museum—not in the birthing room.

## Figures and Tables

**Table 1:** German Mark System Translated into “Centimeters.” Source: Walter Stoeckel, “VII: Die normale Gerburt” [Normal birth], in *Lehrbuch der Geburtshilfe* [Textbook on obstetrics], ed. Walter Stoeckel (Jena: Gustav Fisher, 1920), 109–288 (translations mine).

Object	Unit translation (Stoeckel 1920)	Actual coin size
One-mark coin	2 cm	2.4 cm
Three-mark coin	3 cm	3.3 cm
Five-mark coin	4 cm	3.8 cm
		
Small palm size	6 cm	
Palm size	8 cm	
Full opening	10 cm	

**Table 2:** Familiar Object System Translated into “Centimeters.” Source: Wilhelm Liepmann, “Die Größenbestimmung des äußeren Muttermundes in der Geburt: Ein Vorschlag für Unterricht und Praxis” [The determination of the size of the external cervix during birth: A proposal for teaching and practice], *Zentralbl. Gynaekol*. 45 (1921): 1289 (translations and insertions mine).

A fingertip…………………………………….………	= 1 cm
Wedding ring (average 1.8–2 cm)……………. round	= 2 cm
Ladies’ [wrist] watch (average 2.5–3 cm)……. round	= 3 cm
Men’s [pocket] watch (average 4.5–5 cm)…… round	= 5 cm
Small palm size……………………………..………..	= 6 cm
Palm size……………………………………………..	= 8 cm

## Footnotes

1 Tekoa L. King and Whitney Pinger, “Evidence-Based Practice for Intrapartum Care: The Pearls of Midwifery,” *J. Midwifery Women’s Health* 59, no. 6 (2014): 572–85, quotation on 580, emphases added.

2 Michelle J. K. Osterman, Brady E. Hamilton, Joyce A. Martin, et al., “Births: Final Data for 2020,” *Nat. Vital Stat. Rep*. 70, no. 17 (2022): 30.

3 This definition is currently under debate. For a review on the current diversity of perspectives on how to characterize active labor onset, see Gillian E. Hanley, Sarah Munro, Devon Greyson, et al., “Diagnosing Onset of Labor: A Systematic Review of Definitions in the Research Literature,” *BMC Pregnancy Childbirth* 16, no. 71 (2016): 1–11.

4 American College of Obstetricians and Gynecologists (ACOG) and Society for Maternal-Fetal Medicine (SMFM), “Obstetric Care Consensus No. 1: Safe Prevention of the Primary Cesarean Delivery,” *Obstet. Gyn*. 123 (2014): 693–711; ACOG, “Practice Bulletin No. 205: Vaginal Birth After Cesarean Delivery,” *Obstet. Gyn*. 133, no. 2 (2019): e110–27.

5 Yan Shao, Samuel C. Forster, Evdokia Tsaliki, et al., “Stunted Microbiota and Opportunistic Pathogen Colonization in Caesarean-Section Birth,” *Nature* 574 (2019): 117–21.

6 ACOG and SMFM, “Obstetric Care Consensus” (n. 4), 5, emphases and insertion added.

7 Emanuel A. Friedman, interview by Roberto Romero, “Giants in Obstetrics and Gynecology: A Conversation with Roberto Romero and Emanuel A. Friedman,” *Amer. J. Obstet. Gyn*. (August 2, 2016): suppl. data, video 1, https://doi.org/10.1016/j.ajog.2016.07.034.

8 Emanuel A. Friedman, “Cervimetry: An Objective Method for the Study of Cervical Dilatation in Labor,” *Amer. J. Obstet. Gyn*. 71, no. 6 (1956): 1189–93, quotation on 1189.

9 Ibid., 1189, 1193.

10 To clarify these metrological terms briefly: “Interval-scale” units have equal distances between subsequent points in the scale (e.g., the difference between 2 and 3 cm is the same as the difference between 3 and 4 cm). As such, they permit meaningful averaging of data. On the other hand, “ordinal-scale” units represent only order; the individual units can be closer or farther away from one another. For example, coffee cup sizes of small, medium, and large communicate information about relative sizes, but we would not know whether the difference between the small and medium sizes is the same as the difference between the medium and large sizes. I explore the philosophical details of this episode further in my dissertation, where many of the historical arguments in this paper appear in condensed and/or expanded forms as part B of “Case II: Cervimetry and Epistemic Conduits.” Rebecca L. Jackson, “Measuring ‘Well’: Clinical Measuring Practices and Philosophy of Measurement” (Ph.D. diss., Indiana University Bloomington, 2023).

11 Emanuel A. Friedman, “Evolution of Graphic Analysis of Labor,” *Amer. J. Obstet. Gyn*. 132, no. 7 (1978): 824–27.

12 Emanuel A. Friedman, “Comment: Physiology of Cervical Change over the Course of Labor,” *Eur. J. Obstet. Gyn. Reprod. Biol*. 61 (1995): 179–80.

13 As Felten and von Oertzen have observed, there is nothing inherently abusive or anti-epistemic in bureaucratic processes and methods; indeed, they can be used to “mobilize knowledge about particulars … how to act in specific situations.” There is room for an ethically neutral interpretation of the term “bureaucratized” that I employ here. Sebastian Felten and Christine von Oertzen, “Bureaucracy as Knowledge,” *J. Hist. Knowl*. 1, no. 1 (2020): 1–16, quotation on 7.

14 The changing treatment of cervical dilation data during this period is in line with what Dan Bouk claims is a much broader pattern in American personal data collection between World War II and the 1970s. That is, the inscription of an individual’s “data double” is in service of creating a “data aggregate” at first, and then the individual’s data are located within that data aggregate in an effort to predict and control that individual. Dan Bouk, “The History and Political Economy of Personal Data over the Last Two Centuries in Three Acts,” *Osiris* 32, no. 1 (2017): 85–106.

15 Jacqueline H. Wolf, *Cesarean Section: An American History of Risk, Technology, and Consequence* (Baltimore: Johns Hopkins University Press, 2018), 8, 11–13.

16 Ibid., 99.

17 Helen Feltovich, “Cervical Evaluation from Ancient Medicine to Precision Medicine,” *Obstet. Gyn*. 130 (2017): 51–63.

18 M. Norton Wise, *The Values of Precision* (Princeton, N.J.: Princeton University Press, 1995).

19 Walter Stoeckel, “VII: Die normale Gerburt” [Normal birth], in *Lehrbuch der Geburtshilfe* [Textbook on obstetrics], ed. Walter Stoeckel (Jena: Gustav Fisher, 1920), 109–288, table on 212. This system drops the two-mark unit, probably for not occupying a convenient conversion to a whole number in centimeters that was not already occupied.

20 Wilhelm Liepmann, “Die Größenbestimmung des äußeren Muttermundes in der Geburt: Ein Vorschlag für Unterricht und Praxis” [The determination of the size of the external cervix during birth: A proposal for teaching and practice], *Zentralbl. Gynaekol*. 45 (1921): 1289 (translation mine).

21 Ibid., translation and insertions mine. In this period, a “men’s watch” would most likely have referred to a pocket watch (hence the larger face, larger diameter). A “ladies’ watch” would have referred to a wristwatch (hence the smaller face and diameter).

22 Judith Walzer Leavitt, *Brought to Bed: Childbearing in America, 1750–1950*, 2nd ed. (1986; repr., Oxford: Oxford University Press, 2016), 150.

23 Ibid., 148; Olga McNeile, “Sociological Aspects of Gynecology and Obstetrics,” *Med. Woman’s J*. 29 (1922): 108.

24 See, e.g., Th. Koller and K. Abt, “Das vertical Partogramm als zustäzliche Kontrolle des Geburtsverlaufes” [The vertical parturiogram as additional control of labor], *Gynaecologia* 130 (1950): 419–24.

25 Karl Zimmer, “Die Muttermundseröffnung bei den Schädellagen im Wegzeit-Diagramm,” *Archiv für Gynäkologie* 179, no. 5 (1951): 495.

26 Koller and Abt, “Das vertical Partogramm” (n. 24), 420.

27 Martin S. Pernick, *A Calculus of Suffering: Pain, Professionalism and Anesthesia in Nineteenth-Century America* (New York: Columbia University Press, 1985), has outlined the complexities of nineteenth-century views of pain and its role (or lack thereof) in aiding childbirth. The association between “pain” and “pains” (a common term for contractions at the time) led some to believe pain itself was a required part of the physiological process of birth (46). Others thought pain was a pathological departure from “natural” labor (96). That some practitioners viewed pain as an essential guide is evidenced by primary source excerpts in Leavitt’s *Brought to Bed* (n. 22), which I reference heavily in this section. Leavitt notes in passing that “the progression of [pain] could mark labor’s progress,” but she does not make this point central to her arguments about the changing landscape of childbirth in the nineteenth and twentieth centuries (117). Even in the early to mid-twentieth century, some American doctors (e.g., L. A. Calkins) relied on the German *wehenzahlen* (pain count) method (counting the number of contractions) for assessing progress in the second stage of labor (wherein the cervix is already fully dilated). Lawrence D. Longo, *The Rise of Fetal and Neonatal Physiology: Basic Science to Clinical Care*, 2nd ed. (2013; reis., New York: Springer, 2018), 257.

28 Leavitt, *Brought to Bed* (n. 22), 117; Charles F. Meigs, “Dr. Meigs’ Reply to Professor Simpson’s Letter,” *Med. Examiner Rec. Med. Sci*. 11 (1848): 148–51.

29 Leavitt, *Brought to Bed* (n. 22), 194.

30 Ibid., 206.

31 Ibid., 182.

32 Friedman, “Giants in Obstetrics and Gynecology” (n. 7), video 1.

33 Emanuel A. Friedman, “The Graphic Analysis of Labor,” *Amer. J. Obstet. Gyn*. 68, no. 6 (1954): 1568–75, quotation on 1569.

34 Ibid., 1575.

35 Ibid., 1568.

36 Roberto Romero, “Giants in Obstetrics and Gynecology: A Profile of Emanuel A. Friedman, MD, DMedSci,” *Amer. J. Obstet. Gyn*. 215, no. 4 (2016): 413–14, quotation on 413.

37 Friedman, “Graphic Analysis of Labor” (n. 33).

38 Friedman, “Cervimetry” (n. 8), 1189.

39 Friedman, “Graphic Analysis of Labor” (n. 33), 1569.

40 Goodrich C. Schauffler, “General Summary of the Sessions on Obstetrics of the First American Congress on Obstetrics and Gynecology,” *Amer. J. Obstet. Gyn*. 39, no. 3 (1940): 535–41, quotation on 541.

41 Leroy A. Calkins, “Reproduction in the Older Woman,” *JAMA* 141, no. 10 (1949): 635–38, quotation on 635.

42 Ibid., 638.

43 Emanuel A. Friedman, “Primigravid Labor: A Graphicostatistical Analysis,” *Amer. J. Obstet. Gyn*. 6, no. 6 (1955): 567–89, quotation on 568.

44 Ibid., 568.

45 Friedman, “Graphic Analysis of Labor” (n. 33).

46 Koller and Abt, “Das vertical Partogramm” (n. 24); Zimmer, “Die Muttermundseröffnung” (n. 25).

47 Friedman, “Graphic Analysis of Labor” (n. 33), 1574.

48 Ibid., 1571–72.

49 Ibid., 1571.

50 Ibid., 1571.

51 Ibid., 1571.

52 Ibid., 1572, insertion added.

53 Ibid., 1573.

54 Ibid., 1573.

55 Ibid., 1574.

56 Ibid., 1570.

57 Ibid., 1570.

58 Emanuel A. Friedman, “Notebook, Research in the Graphical Analysis of Labor, Sloane Hospital, 1953,” Emanuel A. Friedman Papers, 1953–1989, Archives & Special Collections, Columbia University Health Sciences Library, backmatter.

59 Friedman, “Primigravid Labor” (n. 43), 570.

60 When the cervix is nearly fully dilated, many practitioners assess dilation negatively, by feeling what remains of the cervical rim from full dilation (now considered 10 cm by convention) and estimating backward, “first as a thin rim, then as an anterior lip that recedes, seemingly reluctantly, to yield full dilatation.” Wayne R. Cohen and Emanuel A. Friedman, “Misguided Guidelines for Managing Labor,” *Amer. J. Obstet. Gyn*. 212, no. 6 (2015): 753–54.

61 Friedman, “Cervimetry” (n. 8), 1189.

62 Ibid., 1189.

63 Ibid., 1190.

64 Ibid., 1189.

65 Ibid., 1189.

66 Ibid., 1190.

67 Ibid., 1191.

68 Ibid., 1191.

69 Ibid., 1191.

70 Ibid., 1191.

71 Ibid., 1190. It does not seem that these X-ray images were taken every time the cervimeter was placed, but simply that images confirmed that the method of placing the cervimeter (with the aid of the Kelly clamp) was generally successful.

72 Ibid., 1190.

73 Ibid., 1190.

74 Ibid., 1191.

75 Ibid., 1193. An anonymous reviewer pointed out that this is an exemplar of the rise of “mechanical objectivity,” or the equating of scientific “objectivity” with the operation of automatic processes (or the regimented use of machines or tools) and the exclusion of individual human influence. Lorraine Daston and Peter Galison, *Objectivity* (New York: Zone Books, 2007).

76 Friedman, “Cervimetry” (n. 8), 1192.

77 By “qualitative” I refer to the S-shaped trend (the pattern of going from linear to concave-up curvature, then linear, then concave-down curvature). This is defined by the change in the sign of the second derivative (see Figure 2) from zero (linear) to positive, to zero, to negative, respectively. Thus, this is a mathematically definable pattern, even if in practice it was being assessed visually without calculation. Yet I use the word “qualitative” to emphasize that what Friedman set out to establish was not one particular set of quantitative thresholds (in centimeters or hours) but rather an overall shape. The “S” could be stretched out for lengthy labors or narrow for speedy labors, and both labors would be considered “normal” under Friedman’s initial intentions for his model.

78 Friedman, “Cervimetry” (n. 8), 1192.

79 Ibid., 1192.

80 Ibid., 1192.

81 Ibid., 1193.

82 Ibid., 1192.

83 Ibid., 1193, insertion added.

84 I thank an anonymous reviewer for pointing out that this is a good example of the complementary nature of two distinct methods of measurement acting as mutual evidential, as discussed by Ian Hacking, *Representing and Intervening: Introductory Topics in the Philosophy of Natural Science* (Cambridge: Cambridge University Press, 1983).

85 Friedman, “Cervimetry” (n. 8), 1193.

86 Ibid., 1193.

87 Emanuel A. Friedman and Lajos I. von Micsky, “Electronic Cervimeter: A Research Instrument for the Study of Cervical Dilatation in Labor,” *Amer. J. Obstet. Gyn*. 87, no. 6 (1963): 789–92, quotation on 791– 92.

88 Friedman retroactively omits the role of the cervimeter when he later recounts his early work (Friedman, “Evolution of Graphic Analysis of Labor” [n. 11]). Perhaps it was not his proudest accomplishment in hindsight, but his cervimeter study was one of the four papers that made up his doctoral thesis arguing for the sigmoidal dilatation-time curve. Emanuel A. Friedman, “Cervimetry: A Study of the Parturient Cervix Uteri with Particular Reference to Dilation” (doctoral thesis, 1959), Emanuel A. Friedman Papers, Archives & Special Collections, Columbia University Health Sciences Library.

89 Friedman, “Comment” (n. 12).

90 Friedman, “Cervimetry” (n. 8), 1193.

91 Ironically, the motivations for creating a cervimeter have shifted from a research tool to support the sufficiency of digital estimation to creating a clinical tool intending to replace the digital method, as if it is not accurate or reliable enough. For an overview of the main types of cervimeters that were designed in the twentieth century, why they failed, and why we are unlikely to ever see the digital method replaced, see Rebecca L. Jackson and Merlin Wassermann, “When Standard Measurement Meets Messy Genitalia: Lessons from 20th Century Phallometry and Cervimetry,” *Stud. Hist. Philos. Sci*. 95 (2022): 37–49, quotation on 45.

92 Ariel L. Zimerman, “The Use of Two-Dimensional (2D) and Three-Dimensional (3D) Ultrasound in the First Stage of Labor,” in *Intrapartum Ultrasonography for Labor Management*, ed. Antonio Malvasi (Berlin: Springer, 2012), 29–40, quotation on 38.

93 Ibid., 29.

94 Even in labor management in the Netherlands and Sweden, countries known for their robust midwifery care systems, the partograph is used as a central tool. See, e.g., Mieneke de Bruin-Kooistra, Marianne P. Amelink-Verburg, Simone E. Buitendijk, et al., “Finding the Right Indicators for Assessing Quality Midwifery Care,” *Int. J. Quality Health Care* 24, no. 3 (2012): 301–10; Ellen L. Tilden, Aaron B. Caughey, Mia Ahlberg, et al., “Latent Phase Duration and Associated Outcomes: A Contemporary, Population-Based Observational Study,” *Amer. J. Obstet. Gyn*. 228, no. 5 (2023): S1025–36.

95 Tina Lavender, Anna Hart, and Rebecca Smyth, “Effect of Partogram Use on Outcomes for Women in Spontaneous Labour at Term,” *Cochrane Database Syst. Rev*. 10, no. 7 (2013): CD005461; Asha R. Dalal and Ameya C. Purandare, “The Partograph in Childbirth: An Absolute Essentiality or a Mere Exercise?,” *J. Obstet. Gyn. India* 68, no. 1 (2017): 3–14.

96 American Nurses Association (ANA), Health Services Administration, Bureau of Quality Assurance, “Guidelines for Review of Nursing Care at the Local Level: Emphasis Given to Professional Standards Review Organizations and the Use of Outcome Criteria in the Review of Nursing Care” (Kansas City, Mo.: ANA, 1976), 80–82.

97 Ibid., 80–81.

98 Leah M. McClimans, “First Person Epidemiological Measures: Vehicles for Patient Centered Care,” *Synthese* 198, no. S10 (2019): 2521–37; Ruth R. Faden and Tom L. Beauchamp, *A History and Theory of Informed Consent* (Oxford: Oxford University Press, 1986).

99 Friedman, “Primigravid Labor” (n. 43), 587.

100 Friedman, “Giants in Obstetrics and Gynecology” (n. 7), video 1.

101 ACOG and SMFM, “Obstetric Care Consensus” (n. 4).

102 Osterman et al., “Births” (n. 2).

103 Cohen and Friedman, “Misguided Guidelines for Managing Labor” (n. 60).

104 Antonio Ragusa, Salvatore Gizzo, Marco Noventa, et al., “Prevention of Primary Caesarean Delivery: Comprehensive Management of Dystocia in Nulliparous Patients at Term,” *Arch. Gyn. Obstet*. 294, no. 4 (2016): 753–61, quotation on 753.

105 Ibid., 759.

106 Ibid., 753.

107 Feltovich, “Cervical Evaluation” (n. 17), 59; Soo Downe, Gillian M. L. Gyte, Hannah G. Dahlen, et al., “Routine Vaginal Examinations for Assessing Progress of Labour to Improve Outcomes for Women and Babies at Term,” *Cochrane Database Syst. Rev*. 7, no. CD010088 (2013): 1–37; Ashley Shepherd, Helen Cheyne, Susan Kennedy, et al., “The Purple Line as a Measure of Labour Progress: A Longitudinal Study,” *BMC Pregnancy Childbirth* 10, no. 54 (2010): 1–7.

108 American Baby and Child Law Centers (ABC Law), “Rewriting the Rules of ‘Normal Labor’: What Does This Mean for Mothers?,” *ABC Birth Injury Blog*, October 20, 2017, https://www.abclawcenters.com/blog/2017/02/23/new-labor-guidelines-decreased-c-section-more-birth-trauma/.

109 The ABC Law blog post was likely written by Jesse M. Reiter and Emily G. Thomas of Reiter & Walsh PC, adapted from their paper “ACOG Replaced the Friedman Curve with Unproven Guidelines: Is Lowering the Caesarean Rate Worth the Rise in Birth Trauma?,” *Amer. Assoc. Justice Birth Trauma Litigation Group Newsl*. (2016).

110 ABC Law, “Rewriting the Rules of ‘Normal Labor’” (n. 108).

111 Ibid., emphasis added.

112 Ibid.

113 Theodore Porter, *Trust in Numbers: The Pursuit of Objectivity in Science and Public Life* (Princeton, N.J.: Princeton University Press, 1995), 195.

114 Ibid., 196.

115 Wolf, *Cesarean Section* (n. 15), 8.

116 Ibid.; Theresa Morris, *Cut It Out: The C-Section Epidemic in America* (New York: New York University Press, 2016). I thank an anonymous reviewer for suggesting that I reference these works to offer a picture of the broader landscape of reasons for C-section rate increases.

117 Dan Bouk, “Women Who Worked with Documents to Rationalize Reproduction,” in *Working with Paper: Gendered Practices in the History of Knowledge*, ed. Carla Bittel, Elaine Leong, and Christine von Oert (Pittsburgh: University of Pittsburgh Press, 2019), 193–207, quotation on 195.

118 Koller and Abt, “Das vertical Partogramm” (n. 24).

119 Zimmer, “Die Muttermundseröffnung” (n. 25).

120 Practitioners are trained to coordinate these assessments with feeling the consistency of their own cheek, nose, and forehead, respectively.

121 In addition to the thoroughly exposed racist and classist beliefs that were codified in pelvic examinations and typification, these same biases also affected perceptions of how (and which) birthing bodies experienced pain. Caroline VanSickle, Kylea L. Liese, and Julienne N. Rutherford, “Textbook Typologies: Challenging the Myth of the Perfect Obstetric Pelvis,” *Anatomical Rec*. 305, no. 4 (2022): 952–67.

122 Friedman, “Notebook” (n. 58).

